# Supplementary material for: Characterisation of the opposing effects of G6PD deficiency on cerebral malaria and severe malarial anaemia
Source: eLife. 2017 Jan 9;6:e15085. doi: 10.7554/eLife.15085 (PMC5222559; doi:10.7554/eLife.15085)
Supplement: Supplementary file 1. — (A) Summary of study designs of contributing partner studies to MalariaGEN Consortial Project 1 (CP1). (B) Genotyped sample distribution. (C) Summary of 65 SNPs selected for analysis and successfully genotyped. (D) G6PD+202 female association test results. (E) G6PD+202 male association test results. (F) G6PD+202 all individuals association test results. (G) G6PD-deficiency score female categorical model female association test results. (H) G6PD-deficiency score categorical model male association test results. (I) G6PD-deficiency score categorical model all individuals association test results. (J) G6PD-deficiency score additive model association test results. DOI: http://dx.doi.org/10.7554/eLife.15085.019 [file elife-15085-supp1.docx]

**Supplementary file 1A-J**

| **Country** | **Cases** | **Control** | **Study Design** |
| --- | --- | --- | --- |
| Gambia | 2,801 | 4,527 | Unmatched Case-Control study. Cases recruited from hospitals in/near Banjul. Controls are cord blood samples collected from labour wards of various hospitals primarily in western division of the country. Other samples from Gambian Biobank blood donors. |
| Mali | 510 | 389 | Matched Case-Control study. Cases recruited from a hospital in Bamako. Controls recruited from community, individually matched to cases by age, ethnicity, place of residence & duration of residence. |
| Burkina Faso | 983 | 816 | Unmatched Case-Control study. Cases recruited from hospitals in Ouagadougou. Controls recruited from rural villages near Ouagoadougou. |
| Ghana  (Navrongo/Noguchi) | 2,459 | 2,129 | Matched Case-control study. Cases recruited from hospitals in Kassena-Nankana District. Controls selected from demographic surveillance system in same district, some frequency & some individually matched to cases by age, gender, location & ethnicity. |
| Ghana  (Kumasi) | 1,923 | 2,326 | Unmatched Case-Control study. Cases recruited from hospitals in Kumasi. Controls are cord blood samples recruited from labour wards in Kumasi. |
| Nigeria | 114 | 88 | Unmatched Case-Control study. Cases recruited from hospitals in Ibadan. Controls are recruited from communities in areas surrounding these hospitals. |
| Cameroon | 914 | 914 | Unmatched Case-Control study. Cases recruited from hospitals/health centres in South-West, Litteral & Central Regions. Controls recruited from schools in South-West Region and blood bank in Central Region. |
| Kenya | 2,741 | 4,183 | Unmatched Case-Control study. Cases recruited from Kilifi District Hospital. Controls recruited from demographic surveillance system representative of area in which cases reside. |
| Tanzania | 501 | 504 | Matched Case-Control study. Cases recruited from a hospital in Muheza. Controls recruited from community, individually matched to cases by ethnicity of at least one parent, electorial ward of residence & age. |
| Malawi | 1,815 | 3,272 | Unmatched Case-Control study. Cases recruited from a hospital in Blantyre. Controls are cord blood samples taken from same hospital. |
| Viet Nam | 1,014 | 27,91 | Matched Case-control study. Cases recruited from a hospital in Ho Chi Minh City & provincial hospitals in Southern Vietnam. Controls recruited from community, individually matched to cases by age, gender, ethnicity & location, & cord blood samples taken from a hospital in Ho Chi Minh City and a hospital in Dong Thap province. |
|  |  |  |  |
| PNG | 658 | 553 | Matched Case-Control study. Cases recruited from main hospital in Madang province. Healthy controls recruited from community, individually matched to cases by ethnicity, age, gender & residence where possible. |
|  |  |  |  |
| **Total** | 16,433 | 22,492 |  |

Supplementary file 1A. Summary of study designs of contributing partner studies to MalariaGEN Consortial Project 1 (CP1). Data includes total numbers of cases and controls collected at each site preceding the filtering process and genotyping as described in Materials and Methods.

|  | Prior to QC |  | QC |  | After QC |
| --- | --- | --- | --- | --- | --- |
| Study site | **Samples Genotyped**  **N (Case/Ctl/Missing)** |  | **Samples Removed N(%)** |  | **Samples Retained**  **N (Case/Ctl/Missing)** |
| Gambia | 6344(2577/3711/56) |  | 504(7.9) |  | 5840(2506/3281/53) |
| Mali | 817(453/345/19) |  | 34(4.2) |  | 783(436/328/19) |
| Burkina Faso | 888(432/456/0) |  | 6(0.7) |  | 882(427/455/0) |
| Ghana (Navrongo) | 6466(2298/3961/207) |  | 87(1.3) |  | 6379(2244/3935/200) |
| Ghana (Kumasi) | 3215(693/512/2010) |  | 1958(60.9) |  | 1257(668/197/392) |
| Nigeria | 160(78/41/41) |  | 2(1.2) |  | 158(77/40/41) |
| Cameroon | 3564(1500/2064/0) |  | 48(1.3) |  | 3516(1489/2027/0) |
| Kenya | 3482(796/2545/141) |  | 44(1.3) |  | 3438(793/2505/140) |
| Tanzania | 1618(867/732/19) |  | 32(2) |  | 1586(846/721/19) |
| Malawi | 818(393/244/181) |  | 17(2.1) |  | 801(383/242/176) |
| Vietnam | 1408(628/580/200) |  | 13(0.9) |  | 1395(622/576/197) |
| Papua New Guinea | 4358(1416/2709/233) |  | 173(4) |  | 4185(1380/2582/223) |
| Total | 33138(12131/17900/3107) |  | 2918(8.8) |  | 30220(11871/16889/1460) |

Supplementary file 1B. Genotyped Sample Distribution. Distribution of genotyped sample prior to quality control (QC) and following QC filters for individual and SNP missingness as described in Materials and Methods.

| SNP (Codon, Alternative name) | Locus | Gene | Base  Change | Functionality | WHO | | Allele Frequency | F_ST_ | Countries  in which SNP is  monomorphic |
| --- | --- | --- | --- | --- | --- | --- | --- | --- | --- |
| rs763737 | 153278307 | *IRAK1* | G/A | IT |  | 0.515(0.029-0.671) | | 0.142 | None |
| rs1734792 | 153341060 | *MECP2* | C/A | IT |  | 0.324(0.163-0.757) | | 0.109 | None |
| rs766420 | 153554404 | *TKTL1* | C/G | IT |  | 0.573(0.269-0.891) | | 0.081 | None |
| rs915941 | 153626649 | *RPL10* | C/A | 5' UTR |  | 0.548(0.122-0.941) | | 0.125 | None |
| rs915942 | 153626738 | *RPL10* | G/A | 5' UTR |  | 0.35(0.059-0.88) | | 0.135 | None |
| rs762513 | 153675171 | *FAM50A* | A/G | IT |  | 0.295(0.179-0.422) | | 0.077 | VN, PNG |
| rs28470352 | 153753490 | *-* | T/A | IG |  | 0.38(0.311-0.557) | | 0.100 | VN, PNG |
| rs61042368 | 153755336 | *-* | G/A | IG |  | 0.118(0.05-0.155) | | 0.025 | VN, PNG |
| rs12389569 | 153757734 | *-* | G/A | IG |  | 0.104(0.054-0.186) | | 0.028 | VN, PNG |
| G6PD_X_b36_153411172 | 153757978 | *-* | C/G | IG |  | 0.003(0.003-0.003) | | n.c. | All except PNG |
| rs12393550 | 153758660 | *-* | G/A | IG |  | 0.354(0.28-0.546) | | 0.091 | VN, PNG |
| rs184533364 | 153759372 | *-* | C/T | IG |  | 0.005(0.005-0.005) | | n.c. | All except VN |
| rs188196644 | 153759426 | *-* | C/A | IG |  | 0.002(0-0.003) | | 0.0002 | All except ML, BF, GH-K, KY |
| G6PD_X_b36_153412734 | 153759540 | *-* | G/A | IG |  | 0.001(0-0.008) | | 0.011 | All except GM, PNG |
| rs181015082 | 153759667 | *G6PD* | G/A | 3' UTR |  | 0.002(0-0.015) | | 0.004 | All except GM, GH-K, VN, PNG |
| CM973154 | 153760261 | *G6PD* | A/C | NS-coding | I | 0.0004 | | n.c. | All except GM |
| rs77214077 | 153760429 | *G6PD* | G/A | S-coding |  | 0.075(0.026-0.135) | | 0.024 | VN, PNG |
| rs72554665 (1376 Canton) | 153760484 | *G6PD* | C/A | NS-coding | II | 0.003(0-0.006) | | 0.003 | All except GM, VN |
| rs2071429 | 153760508 | *G6PD* | G/A | IT |  | 0.177(0.024-0.96) | | 0.653 | Ng |
| rs398123546 | 153760605 | *G6PD* | G/A | NS-coding | II | 0.001(0-0.003) | | 0.002 | All except GM, VN, PNG |
| rs2230037 | 153760654 | *G6PD* | G/A | S-coding |  | 0.273(0.011-0.352) | | 0.037 | None |
| CM067413 | 153760883 | *G6PD* | G/C | NS-coding |  | 0 | | n.c. | All |
| rs2230036 | 153760953 | *G6PD* | C/T | S-coding |  | 0.108(0.061-0.152) | | 0.024 | VN, PNG |
| rs137852342 (1024 Chinese 5) | 153761184 | *G6PD* | G/A | S-coding | III | 0.001(0.001-0.002) | | 0.0001 | All except ML, VN |
| rs76723693 (968) | 153761240 | *G6PD* | T/C | NS-coding | III | 0.037(0-0.069) | | 0.052 | All except GM, ML, BF |
| rs137852327 (871 Viangchan) | 153761337 | *G6PD* | C/T | NS-coding | II | 0.005(0.001-0.013) | | 0.015 | All except GM, VN, PNG |
| rs183394670 | 153761515 | *G6PD* | C/T | IT |  | 0.003(0-0.034) | | 0.031 | All except PNG |
| rs73573478 | 153761564 | *G6PD* | G/A | IT |  | 0.104(0.063-0.155) | | 0.024 | VN, PNG |
| rs5986990 | 153761628 | *G6PD* | G/A | IT |  | 0.374(0.298-0.546) | | 0.099 | VN, PNG |
| rs781934049 | 153761743 | *G6PD* | T/C | IT |  | 0 | | n.c. | All |
| CM014189 | 153761820 | *G6PD* | T/A | NS-coding | II | 0.0005(0.0003-0.0008) | | 0 | All except GM, GH-K,KY |
| rs2515905 | 153762075 | *G6PD* | G/A | IT |  | 0.193(0.054-0.277) | | 0.059 | VN, PNG |
| rs137852328 (680) | 153762340 | *G6PD* | G/T | NS-coding | III | 0.0002 | | n.c. | All except GM |
| rs5986875 | 153762392 | *G6PD* | G/A | IT |  | 0.017(0.001-0.037) | | 0.009 | PNG |
| rs137852330 (592 Coimbra) | 153762605 | *G6PD* | G/A | NS-coding | II | 0.0004(0.0002-0.0004) | | 0 | All except GM, VN |
| rs5030868 (563)  Mediterranean) | 153762634 | *G6PD* | G/A | NS-coding | II | 0.001(0-0.008) | | 0.002 | All except KY, PNG |
| rs5030872 (542) | 153762655 | *G6PD* | A/T | NS-coding | II | 0.008(0-0.014) | | 0.006 | All except GM, ML, BF, GH-K |
| rs137852314 (487 Mahidol) | 153762710 | *G6PD* | C/T | NS-coding | III | 0 | | n.c. | All |
| rs2515904 | 153762771 | *G6PD* | G/C | IT |  | 0.197(0.057-0.298) | | 0.060 | VN,PNG |
| G6PD_X_b36_153416019 | 153762825 | *G6PD* | C/T | IT |  | 0.003(0.003-0.003) | | n.c. | All except PNG |
| CM970547 | 153763462 | *G6PD* | G/A | NS-coding | II | 0.001(0.001-0.001) | | n.c. | All except GM |
| rs78365220 | 153763485 | *G6PD* | A/G | NS-coding | II | 0.003(0-0.037) | | 0.024 | All except GM, PNG |
| rs1050829 (376) | 153763492 | *G6PD* | T/C | NS-coding | IV | 0.385(0.321-0.546) | | 0.103 | VN,PNG |
| rs137852349 (298 Namoru) | 153764211 | *G6PD* | A/G | NS-coding | II | 0 | | n.c. | All |
| rs1050828 (202) | 153764217 | *G6PD* | C/T | NS-coding | III | 0.148(0.027-0.31) | | 0.049 | VN,PNG |
| CM052878 | 153764223 | *G6PD* | A/T | NS-coding | II | 0 | | n.c. | All |
| rs762515 | 153764528 | *G6PD* | T/C | IT |  | 0.397(0.345-0.565) | | 0.107 | VN,PNG |
| rs762516 | 153764663 | *G6PD* | C/T | IT |  | 0.198(0.057-0.298) | | 0.061 | VN,PNG |
| rs73641103 | 153769889 | *G6PD* | G/A | IT |  | 0.015(0.003-0.049) | | 0.006 | VN,PNG |
| rs193062832 | 153771038 | *G6PD* | T/C | IT |  | 0.004(0.001-0.03) | | 0.005 | TZ, VN,PNG |
| rs113492957 | 153773062 | *G6PD* | C/T | IT |  | 0.109(0.076-0.151) | | 0.024 | VN,PNG |
| G6PD_X_b36_153426313 | 153773119 | *G6PD* | G/A | IT |  | 0.001(0-0.007) | | 0.003 | All except GM, CM, MW |
| rs149902811 | 153773160 | *G6PD* | A/C | IT |  | 0.042(0.009-0.082) | | 0.023 | VN,PNG |
| rs145036913 | 153773526 | *G6PD* | A/G | IT |  | 0.006(0.002-0.028) | | 0.009 | GH-N, NG, VN, PNG |
| G6PD_X_b36_153427408 | 153774214 | *G6PD* | T/C | IT |  | 0.077(0.077-0.077) | | n.c. | All except PNG |
| CM950495 | 153774272 | *G6PD* | T/C | NS-coding | I | 0 | | n.c. | All |
| rs111827785 | 153775785 | *G6PD* | C/T | 5' UTR *G6PD* IKBKG |  | 0.574(0.367-1) | | 0.165 | None |
| rs5986992 | 153776107 | *IKBKG* | C/A | 5' UTR *IKBKG* |  | 0.019(0.006-0.041) | | 0.009 | VN,PNG |
| G6PD_X_b36_153429686 | 153776492 | *IKBKG* | G/T | IT |  | 0.005(0.001-0.009) | | 0.002 | GH-N, NG, VN, PNG |
| rs5986997 | 153827549 |  | C/T | IG |  | 0.003(0.001-0.009) | | 0.002 | NG, TZ, VN, PNG |
| rs4898389 | 153827637 |  | G/A | IG |  | 0.189(0.034-0.986) | | 0.624 | None |
| rs5986877 | 153828269 |  | G/C | IG |  | 0.179(0.025-0.989) | | 0.672 | NG |
| rs7879049 | 153829693 |  | A/G | IG |  | 0.31(0.011-0.406) | | 0.051 | None |
| rs7053878 | 153834100 |  | T/A | IG |  | 0.058(0.023-0.857) | | 0.541 | NG |
| rs60030796 | 153836171 |  | A/G | IG |  | 0.076(0.053-0.121) | | 0.023 | VN, PNG |

**Supplementary file 1C. Summary of 65 SNPs selected for analysis and successfully genotyped.** Locus refers to GRCh37, dbSNP137 and Ensembl build 84. Base change refers to ancestral/derived allele, where ancestral allele is assigned from dbSNP137 or, where not available, from human reference sequence Ensembl build 84. WHO refers to grade of G6PD deficiency based on WHO classification scheme. Allele frequency is calculated in population controls with range given across non-monomorphic sites. *IRAK1*, Interleukin 1 Receptor Associated Kinase 1; *MECP2*, Methyl-CpG Binding Protein 2; *TKTL1*, Transketolase-Like 1; RPL10, Ribosomal Protein L10; *FAM50A*, Family With Sequence Similarity 50 Member A ; *IKBKG*, Inhibitor Of Kappa Light Polypeptide Gene Enhancer In B-Cells; IG, Intergenic; IT,Intronic; NS, non synonymous; S, synonymous; UTR, untranslated region; GM Gambia; ML, Mali; BF, Burkina Faso; GH-N, Ghana (Noguchi); GH-K, Ghana (Kumasi); NG Nigeria; CM, Cameroon; KY, Kenya; TZ, Tanzania; MW, Malawi; VN, Vietnam ; PNG, Papua New Guinea.

|  | |  | Controls | | Cerebral Malaria | | |  | Severe Malarial Anaemia | | |  | All Severe Malaria | | | *P*  Hetero-geneity |
| --- | --- | --- | --- | --- | --- | --- | --- | --- | --- | --- | --- | --- | --- | --- | --- | --- |
| **Site** | **Model** | | | **Frequency^a^** | **Frequency^a^** | **OR**  **(95%CI)** | ***P*** |  | **Frequency^a^** | **OR**  **(95%CI)** | ***P*** |  | **Frequency^a^** | **OR**  **(95%CI)** | ***P*** |  |
| Gambia | | A | 0.03(1543/82/2) | | 0.01(391/10/0) | 0.5(0.26-0.97) | 0.04 |  | 0.03(210/11/1) | 1.16(0.64-2.09) | 0.62 |  | 0.02(1141/45/3) | 0.88(0.62-1.25) | 0.47 | 0.09 |
| Gambia | | D | 0.03(1543/82/2) | | 0.01(391/10/0) | 0.5(0.26-0.98) | 0.04 |  | 0.03(210/11/1) | 1.11(0.59-2.08) | 0.76 |  | 0.02(1141/45/3) | 0.83(0.57-1.21) | 0.34 | 0.12 |
| Gambia | | H | 0.03(1543/82/2) | | 0.01(391/10/0) | 0.51(0.26-1.01) | 0.05 |  | 0.03(210/11/1) | 1.03(0.54-1.99) | 0.92 |  | 0.02(1141/45/3) | 0.8(0.54-1.17) | 0.24 | 0.18 |
| Gambia | | R | 0.03(1543/82/2) | | 0.01(391/10/0) | n.c. | 0.97 |  | 0.03(210/11/1) | 3.65(0.32-41.25) | 0.30 |  | 0.02(1141/45/3) | 2.31(0.38-14.16) | 0.37 | 0.98 |
| Mali | | A | 0.16(114/44/3) | | 0.09(26/6/0) | 0.56(0.22-1.43) | 0.22 |  | 0.18(53/23/3) | 1.22(0.72-2.05) | 0.46 |  | 0.16(134/55/3) | 0.96(0.63-1.48) | 0.87 | n.c. |
| Mali | | D | 0.16(114/44/3) | | 0.09(26/6/0) | 0.57(0.21-1.51) | 0.26 |  | 0.18(53/23/3) | 1.17(0.65-2.13) | 0.60 |  | 0.16(134/55/3) | 0.98(0.61-1.57) | 0.92 | n.c. |
| Mali | | H | 0.16(114/44/3) | | 0.09(26/6/0) | 0.61(0.23-1.64) | 0.33 |  | 0.18(53/23/3) | 1.07(0.58-1.98) | 0.82 |  | 0.16(134/55/3) | 1(0.62-1.61) | 0.99 | n.c. |
| Mali | | R | 0.16(114/44/3) | | 0.09(26/6/0) | n.c. | 0.99 |  | 0.18(53/23/3) | 2.04(0.4-10.46) | 0.39 |  | 0.16(134/55/3) | 0.78(0.15-3.95) | 0.76 | n.c. |
| Burkina | | A | 0.15(252/89/7) | | 0.26(25/21/2) | 1.95(1.15-3.29) | 1.26E-02 |  | 0.22(13/5/2) | 1.55(0.72-3.36) | 0.26 |  | 0.18(242/116/10) | 1.27(0.95-1.69) | 0.10 | n.c. |
| Faso | | D | 0.15(252/89/7) | | 0.26(25/21/2) | 2.23(1.2-4.13) | 1.07E-02 |  | 0.22(13/5/2) | 1.31(0.5-3.38) | 0.58 |  | 0.18(242/116/10) | 1.31(0.95-1.8) | 0.10 | n.c. |
| Burkin.c.Faso | | H | 0.15(252/89/7) | | 0.26(25/21/2) | 2.1(1.13-3.92) | 0.02 |  | 0.22(13/5/2) | 0.9(0.32-2.56) | 0.85 |  | 0.18(242/116/10) | 1.28(0.92-1.78) | 0.15 | n.c. |
| Burkin.c.Faso | | R | 0.15(252/89/7) | | 0.26(25/21/2) | 1.91(0.39-9.49) | 0.43 |  | 0.22(13/5/2) | 4.89(0.95-25.25) | 0.06 |  | 0.18(242/116/10) | 1.37(0.51-3.69) | 0.53 | n.c. |
| Ghana | | A | 0.19(56/32/1) | | 0.23(7/3/1) | 1.38(0.41-4.64) | 0.60 |  | 0.24(57/26/9) | 1.48(0.87-2.53) | 0.15 |  | 0.2(188/89/13) | 1.12(0.72-1.75) | 0.62 | 0.99 |
| (Noguchi)c. | | D | 0.19(56/32/1) | | 0.23(7/3/1) | 0.98(0.25-3.77) | 0.98 |  | 0.24(57/26/9) | 1.14(0.61-2.15) | 0.68 |  | 0.2(188/89/13) | 0.97(0.58-1.61) | 0.89 | 0.98 |
| Ghan.c. | | H | 0.19(56/32/1) | | 0.23(7/3/1) | 0.63(0.15-2.68) | 0.53 |  | 0.24(57/26/9) | 0.72(0.38-1.39) | 0.33 |  | 0.2(188/89/13) | 0.8(0.48-1.33) | 0.38 | 0.93 |
| Ghan.c. | | R | 0.19(56/32/1) | | 0.23(7/3/1) | n.c. | 0.99 |  | 0.24(57/26/9) | 32.8(1.98-543.44) | 1.48E-02 |  | 0.2(188/89/13) | 6.79(0.74-61.96) | 0.09 | 0.93 |
| Ghana | | A | 0.19(644/302/32) | | 0.14(84/28/2) | 0.76(0.5-1.16) | 0.20 |  | 0.21(165/81/13) | 1.2(0.92-1.57) | 0.18 |  | 0.19(454/211/27) | 1.05(0.87-1.26) | 0.63 | 0.03 |
| (Kumasi) | | D | 0.19(644/302/32) | | 0.14(84/28/2) | 0.74(0.46-1.19) | 0.21 |  | 0.21(165/81/13) | 1.23(0.9-1.68) | 0.20 |  | 0.19(454/211/27) | 1.05(0.84-1.3) | 0.68 | 0.05 |
| Ghan.c. | | H | 0.19(644/302/32) | | 0.14(84/28/2) | 0.77(0.47-1.25) | 0.29 |  | 0.21(165/81/13) | 1.19(0.86-1.64) | 0.30 |  | 0.19(454/211/27) | 1.03(0.82-1.29) | 0.79 | 0.16 |
| Ghan.c. | | R | 0.19(644/302/32) | | 0.14(84/28/2) | 0.64(0.15-2.71) | 0.54 |  | 0.21(165/81/13) | 1.33(0.61-2.89) | 0.47 |  | 0.19(454/211/27) | 1.12(0.64-1.97) | 0.69 | 0.16 |
| Nigeria | | A | 0.38(7/12/2) | | 0.25(1/1/0) | 0.49(0.04-6.16) | 0.58 |  | 0.38(2/1/1) | 1.21(0.2-7.42) | 0.84 |  | 0.21(21/7/3) | 0.45(0.19-1.1) | 0.08 | n.c. |
| Nigeria | | D | 0.38(7/12/2) | | 0.25(1/1/0) | 0.55(0.03-10.37) | 0.69 |  | 0.38(2/1/1) | 0.53(0.05-5.23) | 0.58 |  | 0.21(21/7/3) | 0.24(0.07-0.78) | 0.02 | n.c. |
| Nigeria | | H | 0.38(7/12/2) | | 0.25(1/1/0) | 0.89(0.05-16.66) | 0.94 |  | 0.38(2/1/1) | 0.17(0.01-2.77) | 0.21 |  | 0.21(21/7/3) | 0.22(0.07-0.73) | 0.01 | n.c. |
| Nigeria | | R | 0.38(7/12/2) | | 0.25(1/1/0) | n.c. | 1.00 |  | 0.38(2/1/1) | 7.5(0.32-173.28) | 0.21 |  | 0.21(21/7/3) | 1.07(0.16-7.22) | 0.94 | n.c. |
| Cameroon | | A | 0.1(131/25/3) | | 0.03(18/1/0) | 0.22(0.03-1.7) | 0.15 |  | 0.15(31/8/2) | 1.34(0.66-2.71) | 0.42 |  | 0.09(234/47/3) | 0.82(0.51-1.32) | 0.41 | 0.08 |
| Cameroon | | D | 0.1(131/25/3) | | 0.03(18/1/0) | 0.21(0.03-1.69) | 0.14 |  | 0.15(31/8/2) | 1.27(0.54-2.99) | 0.58 |  | 0.09(234/47/3) | 0.83(0.49-1.41) | 0.48 | 0.09 |
| Cameroon | | H | 0.1(131/25/3) | | 0.03(18/1/0) | 0.24(0.03-1.93) | 0.18 |  | 0.15(31/8/2) | 1.07(0.43-2.68) | 0.88 |  | 0.09(234/47/3) | 0.87(0.5-1.5) | 0.61 | 0.19 |
| Cameroon | | R | 0.1(131/25/3) | | 0.03(18/1/0) | n.c. | 0.99 |  | 0.15(31/8/2) | 2.73(0.38-19.32) | 0.32 |  | 0.09(234/47/3) | 0.55(0.1-3.05) | 0.50 | 0.99 |
| Kenya | | A | 0.19(1248/634/62) | | 0.19(289/135/17) | 0.99(0.81-1.2) | 0.90 |  | 0.18(49/20/3) | 0.9(0.57-1.41) | 0.63 |  | 0.18(734/309/37) | 0.9(0.78-1.04) | 0.14 | 0.74 |
| Kenya | | D | 0.19(1248/634/62) | | 0.19(289/135/17) | 0.95(0.76-1.18) | 0.62 |  | 0.18(49/20/3) | 0.82(0.49-1.37) | 0.45 |  | 0.18(734/309/37) | 0.85(0.72-1.01) | 0.06 | 0.63 |
| Kenya | | H | 0.19(1248/634/62) | | 0.19(289/135/17) | 0.9(0.72-1.14) | 0.39 |  | 0.18(49/20/3) | 0.76(0.45-1.31) | 0.33 |  | 0.18(734/309/37) | 0.83(0.7-0.98) | 0.03 | 0.55 |
| Kenya | | R | 0.19(1248/634/62) | | 0.19(289/135/17) | 1.32(0.75-2.32) | 0.34 |  | 0.18(49/20/3) | 1.41(0.43-4.64) | 0.57 |  | 0.18(734/309/37) | 1.13(0.74-1.74) | 0.57 | 0.82 |
| Tanzania | | A | 0.21(154/86/10) | | 0.21(11/5/1) | 0.87(0.35-2.14) | 0.76 |  | 0.13(56/13/3) | 0.51(0.29-0.89) | 0.02 |  | 0.17(141/49/10) | 0.76(0.53-1.07) | 0.12 | n.c. |
| Tanzania | | D | 0.21(154/86/10) | | 0.21(11/5/1) | 0.76(0.27-2.17) | 0.61 |  | 0.13(56/13/3) | 0.4(0.21-0.74) | 3.94E-03 |  | 0.17(141/49/10) | 0.62(0.41-0.94) | 0.02 | n.c. |
| Tanzania | | H | 0.21(154/86/10) | | 0.21(11/5/1) | 0.68(0.23-2.05) | 0.50 |  | 0.13(56/13/3) | 0.35(0.18-0.68) | 2.14E-03 |  | 0.17(141/49/10) | 0.55(0.35-0.85) | 6.79E-03 | n.c. |
| Tanzania | | R | 0.21(154/86/10) | | 0.21(11/5/1) | 1.56(0.17-14.3) | 0.69 |  | 0.13(56/13/3) | 1.25(0.3-5.18) | 0.76 |  | 0.17(141/49/10) | 1.55(0.58-4.13) | 0.38 | n.c. |
| Malawi | | A | 0.2(784/395/46) | | 0.18(293/127/15) | 0.89(0.73-1.09) | 0.27 |  | 0.23(37/24/3) | 1.25(0.82-1.9) | 0.31 |  | 0.19(440/210/23) | 0.95(0.8-1.13) | 0.58 | 0.15 |
| Malawi | | D | 0.2(784/395/46) | | 0.18(293/127/15) | 0.87(0.69-1.1) | 0.24 |  | 0.23(37/24/3) | 1.31(0.79-2.19) | 0.29 |  | 0.19(440/210/23) | 0.95(0.78-1.16) | 0.62 | 0.14 |
| Malawi | | H | 0.2(784/395/46) | | 0.18(293/127/15) | 0.88(0.69-1.12) | 0.29 |  | 0.23(37/24/3) | 1.28(0.76-2.15) | 0.35 |  | 0.19(440/210/23) | 0.97(0.79-1.18) | 0.73 | 0.18 |
| Malawi | | R | 0.2(784/395/46) | | 0.18(293/127/15) | 0.91(0.5-1.65) | 0.75 |  | 0.23(37/24/3) | 1.25(0.38-4.14) | 0.72 |  | 0.19(440/210/23) | 0.9(0.54-1.5) | 0.68 | 0.62 |
| All | | A | 0.15(4933/1701/168) | | 0.14(1145/337/38) | 0.91(0.81-1.03) | 0.15 |  | 0.16(673/212/40) | 1.11(0.95-1.29) | 0.19 |  | 0.14(3729/1138/132) | 0.95(0.88-1.03) | 0.21 | 1.14E-02 |
| All | | D | 0.15(4933/1701/168) | | 0.14(1145/337/38) | 0.88(0.77-1.01) | 0.08 |  | 0.16(673/212/40) | 1.04(0.87-1.24) | 0.68 |  | 0.14(3729/1138/132) | 0.92(0.84-1.01) | 0.07 | 0.03 |
| All | | H | 0.15(4933/1701/168) | | 0.14(1145/337/38) | 0.87(0.75-1) | 0.05 |  | 0.16(673/212/40) | 0.93(0.77-1.13) | 0.47 |  | 0.14(3729/1138/132) | 0.9(0.82-0.99) | 0.03 | 0.16 |
| All | | R | 0.15(4933/1701/168) | | 0.14(1145/337/38) | 1.06(0.73-1.54) | 0.75 |  | 0.16(673/212/40) | 1.86(1.23-2.81) | 3.40E-03 |  | 0.14(3729/1138/132) | 1.13(0.88-1.45) | 0.33 | 0.04 |

Supplementary file 1D. G6PD+202 Female Association Test Results. Frequency of the derived (D) allele at G6PD+202 (rs1050828) in female control and case individuals and odds ratios (ORs), 95% confidence intervals (95% CI) and *P*-values (*P*) for association of the derived allele with severe malaria and sub-types cerebral malaria and severe malarial anaemia additive (A), dominant (D), heterozygous (H) and recessive (R) models for fixed effects at all study sites combined and separately by site. *P*-values for heterogeneity of effect of severe malarial anaemia and cerebral malaria are also shown. Results are adjusted for sickle-cell trait, ethnicity and sex. Sites at which G6PD+202 was monomorphic (Vietnam and Papua New Guinea) were excluded from this analysis. ^a^ Derived allele frequency and counts of wild-type homozygotes, heterozygotes and derived homozygotes respectively. n.c., not calculated.

|  | |  | Controls | Cerebral Malaria | | |  | Severe Malarial Anaemia | | |  | All Severe Malaria | | | | *P*  Hetero-geneity |
| --- | --- | --- | --- | --- | --- | --- | --- | --- | --- | --- | --- | --- | --- | --- | --- | --- |
| **Site** | **Model** | | **Frequency^a^** | **Frequency^a^** | **OR**  **(95%CI)** | ***P*** |  | **Frequency^a^** | **OR**  **(95%CI)** | ***P*** |  | **Frequency^a^** | **OR**  **(95%CI)** | ***P*** | |  |
| Gambia | | M | 0.03(1599/48) | 0.03(402/11) | 0.91(0.46-1.81) | 0.79 |  | 0.03(239/8) | 1.12(0.52-2.43) | 0.77 |  | 0.02(1279/31) | 0.83(0.52-1.34) | 0.45 | 0.78 | |
| Mali | | M | 0.17(138/29) | 0.07(43/3) | 0.29(0.08-1.06) | 0.06 |  | 0.24(78/25) | 1.41(0.75-2.66) | 0.29 |  | 0.19(197/47) | 1.03(0.6-1.74) | 0.93 | 0.02 | |
| BurkinaFaso | | M | 0.14(317/51) | 0.07(53/4) | 0.45(0.16-1.31) | 0.14 |  | 0.22(14/4) | 1.71(0.54-5.41) | 0.36 |  | 0.15(403/72) | 1.09(0.74-1.62) | 0.67 | NA | |
| Ghana (Noguchi) | | M | 0.14(93/15) | 0.1(9/1) | 0.58(0.06-5.14) | 0.62 |  | 0.24(115/36) | 1.91(0.97-3.76) | 0.06 |  | 0.22(293/84) | 1.73(0.94-3.17) | 0.08 | 0.29 | |
| Ghana (Kumasi) | | M | 0.18(847/186) | 0.16(98/18) | 0.79(0.45-1.39) | 0.42 |  | 0.25(218/71) | 1.32(0.93-1.86) | 0.12 |  | 0.2(636/159) | 1.08(0.84-1.38) | 0.56 | 0.04 | |
| Nigeria | | M | 0.17(15/3) | 0(4/0) | n.c. | n.c. |  | 0.33(2/1) | 3(0.14-64.25) | 0.48 |  | 0.09(42/4) | 0.33(0.06-1.76) | 0.20 | NA | |
| Cameroon | | M | 0.12(369/48) | 0.1(18/2) | 0.93(0.21-4.17) | 0.92 |  | 0.2(32/8) | 2.57(1.08-6.09) | 0.03 |  | 0.13(291/44) | 1.31(0.83-2.06) | 0.25 | 0.22 | |
| Kenya | | M | 0.19(1608/375) | 0.16(385/72) | 0.84(0.64-1.12) | 0.24 |  | 0.33(58/28) | 2.22(1.38-3.55) | 9.22E-04 |  | 0.2(921/229) | 1.12(0.93-1.36) | 0.23 | 2.59E-04 | |
| Tanzania | | M | 0.2(165/40) | 0.18(14/3) | 0.97(0.25-3.76) | 0.96 |  | 0.15(91/16) | 0.83(0.42-1.61) | 0.58 |  | 0.15(194/33) | 0.7(0.41-1.19) | 0.19 | 0.87 | |
| Malawi | | M | 0.2(1071/266) | 0.18(357/79) | 0.88(0.66-1.16) | 0.36 |  | 0.29(46/19) | 1.64(0.94-2.85) | 0.08 |  | 0.22(551/151) | 1.09(0.87-1.36) | 0.47 | 0.04 | |
| All | | M | 0.15(6207/1058)^b^ | 0.12(1379/193) | 0.82(0.69-0.98) | 0.03 |  | - | - | - |  | - | - | - | 1.09E-07 | |
|  | | M | 0.15(6222/1061) | - | - | - |  | 0.19(893/216) | 1.48(1.22-1.8) | 6.58E-05 |  | 0.15(4807/854) | 1.08(0.97-1.2) | 0.14 | - | |

Supplementary file 1E. G6PD+202 Male Association Test Results. Frequency of the derived (D) allele at G6PD+202 (rs1050828) in male control and case individuals and odds ratios (ORs), 95% confidence intervals (95% CI) and *P*-values (*P*) are presented for association of the derived allele with severe malaria and sub-types cerebral malaria and severe malarial anaemia under a hemizygous (M) model for fixed effects at all study sites combined and separately by site. *P*-values for heterogeneity of effect of severe malarial anaemia and cerebral malaria are also shown. Results are adjusted for sickle-cell trait, ethnicity and sex. Sites at which G6PD+202 was monomorphic (Vietnam and Papua New Guinea) were excluded from this analysis. ^a^ Derived allele frequency and counts of wild-type and derived hemizygotes respectively. ^b^ Nigeria is excluded from the combined analysis for cerebral malaria. n.c., not calculated.

Supplementary file 1F. G6PD+202 All Individuals Association Test Results. Frequency of the derived (D) allele at G6PD+202 (rs1050828) in all control and case individuals and odds ratios (ORs), 95% confidence intervals (95% CI) and *P*-values (*P*) are presented for association of the derived allele with severe malaria and sub-types cerebral malaria and severe malarial anaemia under additive (A), dominant (D) and recessive (R) models for fixed effects at all study sites combined and separately by site. *P*-values for heterogeneity of effect of severe malarial anaemia and cerebral malaria are also shown. Results are adjusted for sickle-cell trait, ethnicity and sex. Sites at which G6PD+202 was monomorphic (Vietnam and Papua New Guinea) were excluded from this analysis. ^a^ Derived allele frequency and counts of wild-type homozygotes, heterozygotes and derived homozygotes where male hemizygotes are counted as female homozygotes. n.c., not calculated.

|  |  | Controls | Cerebral Malaria | | |  | Severe Malarial Anaemia | | |  | All Severe Malaria | | | *P*  Hetero-geneity |
| --- | --- | --- | --- | --- | --- | --- | --- | --- | --- | --- | --- | --- | --- | --- |
| **Site** | **Model** | **Frequency^a^** | **Frequency^a^** | **OR**  **(95%CI)** | ***P*** |  | **Frequency^a^** | **OR**  **(95%CI)** | ***P*** |  | **Frequency^a^** | **OR**  **(95%CI)** | ***P*** |  |
| Gambia | A | 0.03(3142/82/50) | 0.02(793/10/11) | 0.81(0.59-1.12) | 0.20 |  | 0.03(449/11/9) | 1.09(0.79-1.51) | 0.59 |  | 0.02(2420/45/34) | 0.9(0.74-1.1) | 0.31 | 0.24 |
| Gambia | D | 0.03(3142/82/50) | 0.02(793/10/11) | 0.66(0.41-1.06) | 0.09 |  | 0.03(449/11/9) | 1.12(0.69-1.83) | 0.65 |  | 0.02(2420/45/34) | 0.83(0.62-1.12) | 0.23 | 0.15 |
| Gambia | R | 0.03(3142/82/50) | 0.02(793/10/11) | 0.87(0.45-1.7) | 0.68 |  | 0.03(449/11/9) | 1.22(0.58-2.57) | 0.59 |  | 0.02(2420/45/34) | 0.89(0.56-1.41) | 0.63 | 0.53 |
| Mali | A | 0.17(252/44/32) | 0.07(69/6/3) | 0.56(0.33-0.96) | 0.04 |  | 0.22(131/23/28) | 1.2(0.92-1.56) | 0.18 |  | 0.18(331/55/50) | 1.02(0.82-1.27) | 0.89 | 4.25E-03 |
| Mali | D | 0.17(252/44/32) | 0.07(69/6/3) | 0.45(0.21-0.97) | 0.04 |  | 0.22(131/23/28) | 1.28(0.84-1.95) | 0.26 |  | 0.18(331/55/50) | 1.03(0.73-1.45) | 0.88 | 5.72E-03 |
| Mali | R | 0.17(252/44/32) | 0.07(69/6/3) | 0.3(0.08-1.13) | 0.08 |  | 0.22(131/23/28) | 1.49(0.84-2.65) | 0.17 |  | 0.18(331/55/50) | 1.03(0.63-1.68) | 0.91 | 1.28E-02 |
| Burkina- | A | 0.14(569/89/58) | 0.13(78/21/6) | 1.04(0.77-1.42) | 0.78 |  | 0.22(27/5/6) | 1.39(0.86-2.23) | 0.18 |  | 0.16(645/116/82) | 1.11(0.94-1.31) | 0.21 | NA |
| Faso | D | 0.14(569/89/58) | 0.13(78/21/6) | 1.32(0.83-2.11) | 0.25 |  | 0.22(27/5/6) | 1.45(0.69-3.06) | 0.33 |  | 0.16(645/116/82) | 1.21(0.94-1.56) | 0.13 | NA |
| Burkin.c.Faso | R | 0.14(569/89/58) | 0.13(78/21/6) | 0.62(0.25-1.53) | 0.30 |  | 0.22(27/5/6) | 2.28(0.85-6.12) | 0.10 |  | 0.16(645/116/82) | 1.13(0.78-1.63) | 0.53 | NA |
| Ghana | A | 0.16(149/32/16) | 0.14(16/3/2) | 1(0.47-2.12) | 0.99 |  | 0.24(172/26/45) | 1.39(1.05-1.84) | 0.02 |  | 0.21(481/89/97) | 1.24(0.98-1.57) | 0.07 | 0.40 |
| (Noguchi)c. | D | 0.16(149/32/16) | 0.14(16/3/2) | 0.88(0.3-2.61) | 0.82 |  | 0.24(172/26/45) | 1.43(0.92-2.24) | 0.11 |  | 0.21(481/89/97) | 1.23(0.85-1.78) | 0.27 | 0.46 |
| Ghan.c. | R | 0.16(149/32/16) | 0.14(16/3/2) | 1.34(0.27-6.77) | 0.72 |  | 0.24(172/26/45) | 2.52(1.33-4.76) | 4.55E-03 |  | 0.21(481/89/97) | 1.93(1.1-3.39) | 0.02 | 0.42 |
| Ghana | A | 0.18(1491/302/218) | 0.15(182/28/20) | 0.85(0.67-1.07) | 0.17 |  | 0.23(383/81/84) | 1.16(1.01-1.34) | 0.04 |  | 0.2(1090/211/186) | 1.04(0.94-1.16) | 0.44 | 3.58E-03 |
| (Kumasi)c. | D | 0.18(1491/302/218) | 0.15(182/28/20) | 0.76(0.53-1.09) | 0.14 |  | 0.23(383/81/84) | 1.27(1-1.6) | 0.05 |  | 0.2(1090/211/186) | 1.06(0.9-1.25) | 0.47 | 3.97E-03 |
| Ghan.c. | R | 0.18(1491/302/218) | 0.15(182/28/20) | 0.77(0.46-1.29) | 0.32 |  | 0.23(383/81/84) | 1.32(0.96-1.81) | 0.09 |  | 0.2(1090/211/186) | 1.09(0.86-1.37) | 0.48 | 1.31E-02 |
| Nigeria | A | 0.24(22/12/5) | 0.08(5/1/0) | 0.32(0.07-1.36) | 0.12 |  | 0.35(4/1/2) | 1.65(0.41-6.55) | 0.48 |  | 0.13(63/7/7) | 0.52(0.28-0.96) | 0.04 | NA |
| Nigeria | D | 0.24(22/12/5) | 0.08(5/1/0) | 0.27(0.03-2.45) | 0.25 |  | 0.35(4/1/2) | 1.11(0.12-9.91) | 0.92 |  | 0.13(63/7/7) | 0.27(0.1-0.72) | 9.05E-03 | NA |
| Nigeria | R | 0.24(22/12/5) | 0.08(5/1/0) | 0(0-0) | 0.00 |  | 0.35(4/1/2) | 5.33(0.53-53.41) | 0.15 |  | 0.13(63/7/7) | 0.57(0.16-2.06) | 0.39 | NA |
| Cameroon | A | 0.11(500/25/51) | 0.08(36/1/2) | 0.71(0.3-1.65) | 0.42 |  | 0.18(63/8/10) | 1.52(1.05-2.21) | 0.03 |  | 0.12(525/47/47) | 1.08(0.88-1.33) | 0.45 | 0.09 |
| Cameroon | D | 0.11(500/25/51) | 0.08(36/1/2) | 0.48(0.14-1.68) | 0.25 |  | 0.18(63/8/10) | 1.76(0.94-3.31) | 0.08 |  | 0.12(525/47/47) | 1.1(0.77-1.57) | 0.59 | 0.06 |
| Cameroon | R | 0.11(500/25/51) | 0.08(36/1/2) | 0.8(0.18-3.54) | 0.77 |  | 0.18(63/8/10) | 2.57(1.17-5.66) | 0.02 |  | 0.12(525/47/47) | 1.24(0.79-1.93) | 0.35 | 0.17 |
| Kenya | A | 0.19(2856/634/437) | 0.17(674/135/89) | 0.94(0.84-1.06) | 0.31 |  | 0.28(107/20/31) | 1.31(1.06-1.63) | 1.38E-02 |  | 0.19(1655/309/266) | 1.01(0.93-1.09) | 0.87 | 0.00 |
| Kenya | D | 0.19(2856/634/437) | 0.17(674/135/89) | 0.91(0.76-1.08) | 0.27 |  | 0.28(107/20/31) | 1.35(0.93-1.95) | 0.11 |  | 0.19(1655/309/266) | 0.96(0.85-1.08) | 0.48 | 0.04 |
| Kenya | R | 0.19(2856/634/437) | 0.17(674/135/89) | 0.92(0.71-1.18) | 0.51 |  | 0.28(107/20/31) | 2.05(1.34-3.13) | 9.32E-04 |  | 0.19(1655/309/266) | 1.13(0.95-1.34) | 0.17 | 4.92E-04 |
| Tanzania | A | 0.2(319/86/50) | 0.19(25/5/4) | 0.94(0.54-1.61) | 0.81 |  | 0.14(147/13/19) | 0.76(0.57-1.03) | 0.07 |  | 0.15(335/49/43) | 0.8(0.65-0.99) | 0.04 | 0.48 |
| Tanzania | D | 0.2(319/86/50) | 0.19(25/5/4) | 0.84(0.37-1.9) | 0.67 |  | 0.14(147/13/19) | 0.55(0.35-0.86) | 9.31E-03 |  | 0.15(335/49/43) | 0.64(0.46-0.89) | 8.25E-03 | 0.34 |
| Tanzania | R | 0.2(319/86/50) | 0.19(25/5/4) | 1.03(0.33-3.24) | 0.96 |  | 0.14(147/13/19) | 0.87(0.48-1.58) | 0.65 |  | 0.15(335/49/43) | 0.84(0.53-1.34) | 0.47 | 0.82 |
| Malawi | A | 0.2(1855/395/312) | 0.18(650/127/94) | 0.92(0.82-1.04) | 0.17 |  | 0.27(83/24/22) | 1.27(1.01-1.6) | 0.04 |  | 0.21(991/210/174) | 1.01(0.92-1.11) | 0.77 | 1.12E-02 |
| Malawi | D | 0.2(1855/395/312) | 0.18(650/127/94) | 0.87(0.73-1.05) | 0.14 |  | 0.27(83/24/22) | 1.45(0.99-2.13) | 0.05 |  | 0.21(991/210/174) | 1.01(0.87-1.17) | 0.92 | 1.38E-02 |
| Malawi | R | 0.2(1855/395/312) | 0.18(650/127/94) | 0.88(0.69-1.14) | 0.34 |  | 0.27(83/24/22) | 1.56(0.95-2.55) | 0.08 |  | 0.21(991/210/174) | 1.05(0.86-1.29) | 0.62 | 0.04 |
| All | A | 0.15(11155/1701/1229) | 0.13(2528/337/231) | 0.91(0.85-0.97) | 7.22E-03 |  | 0.18(1566/212/256) | 1.18(1.09-1.28) | 7.48E-05 |  | 0.15(8536/1138/986) | 1.01(0.97-1.06) | 0.65 | 3.99E-09 |
| All | D | 0.15(11155/1701/1229) | 0.13(2528/337/231) | 0.86(0.77-0.95) | 4.54E-03 |  | 0.18(1566/212/256) | 1.21(1.06-1.38) | 5.44E-03 |  | 0.15(8536/1138/986) | 0.98(0.92-1.05) | 0.58 | 1.56E-07 |
| All | R | 0.15(11155/1701/1229) | 0.13(2528/337/231) | 0.86(0.74-1) | 0.06 |  | 0.18(1566/212/256) | 1.53(1.29-1.82) | 1.19E-06 |  | 0.15(8536/1138/986) | 1.09(0.99-1.2) | 0.08 | 1.45E-08 |

|  | | G6PDd  Score | | Controls | | Severe Malaria | | | | |  | | Cerebral Malaria | | | | |  | Severe Malarial Anemia | | |
| --- | --- | --- | --- | --- | --- | --- | --- | --- | --- | --- | --- | --- | --- | --- | --- | --- | --- | --- | --- | --- | --- |
|  | |  |  | **N(%)** | | **N(%)** | | **OR (95%CI)** | ***P^a^*** | |  | | **N(%)** | | **OR (95%CI)** | | ***P^a^*** |  | **N(%)** | **OR (95%CI)** | ***P^a^*** |
| Gambia | 0 | | 766(46.9) | | 589(49.3) | | - | | | ***2.83E-03*** | |  | | 202(50.4) | | - | ***7.18E-03*** |  | 98(43.9) | - | ***0.06*** |
| Gambia | (0-25) | | 520(31.9) | | 403(33.8) | | 1.03(0.86-1.22) | | | 0.77 | |  | | 147(36.7) | | 1.1(0.86-1.41) | 0.44 |  | 75(33.6) | 1.17(0.84-1.62) | 0.35 |
|  | [25-50) | | 310(19) | | 175(14.7) | | 0.75(0.6-0.94) | | | 1.17E-02 | |  | | 48(12) | | 0.6(0.43-0.85) | 4.04E-03 |  | 42(18.8) | 1.09(0.74-1.61) | 0.67 |
|  | [50-85) | | 35(2.1) | | 21(1.8) | | 0.89(0.5-1.58) | | | 0.69 | |  | | 4(1) | | 0.48(0.17-1.38) | 0.17 |  | 6(2.7) | 1.51(0.61-3.76) | 0.37 |
| Gambia | ≥85 | | 1(0.1) | | 6(0.5) | | 13.54(1.46-125.83) | | | 0.02 | |  | | 0(0) | | n.c | n.c |  | 2(0.9) | 54.99(2.85-1061.08) | 7.97E-03 |
| Mali | 0 | | 52(32.3) | | 63(32.8) | | - | | | ***0.95*** | |  | | 10(31.2) | | - | ***0.48*** |  | 24(30.4) | - | ***0.88*** |
| Mali | (0-25) | | 59(36.6) | | 68(35.4) | | 0.93(0.54-1.58) | | | 0.78 | |  | | 16(50) | | 1.19(0.47-2.99) | 0.71 |  | 26(32.9) | 0.93(0.46-1.87) | 0.84 |
|  | [25-50) | | 46(28.6) | | 57(29.7) | | 0.94(0.54-1.64) | | | 0.84 | |  | | 5(15.6) | | 0.51(0.16-1.68) | 0.27 |  | 26(32.9) | 1.19(0.59-2.41) | 0.63 |
|  | [50-85) | | 4(2.5) | | 4(2.1) | | 0.66(0.15-2.84) | | | 0.57 | |  | | 1(3.1) | | 1.14(0.1-12.85) | 0.92 |  | 3(3.8) | 1.41(0.29-6.93) | 0.68 |
| Mali | ≥85 | | 0(0) | | 0(0) | | n.c | | | n.c. | |  | | 0(0) | | n.c | n.c. |  | 0(0) | n.c | n.c. |
| Burkina Faso | 0 | | 111(31.7) | | 130(35.3) | | - | | | ***0.09*** | |  | | 12(25) | | - | ***0.11*** |  | 7(35) | - | ***0.48*** |
| Burkina Faso | (0-25) | | 139(39.7) | | 110(29.9) | | 0.69(0.48-0.99) | | | 0.04 | |  | | 13(27.1) | | 0.87(0.38-2) | 0.75 |  | 6(30) | 0.69(0.22-2.12) | 0.52 |
|  | [25-50) | | 91(26) | | 118(32.1) | | 1.07(0.74-1.56) | | | 0.71 | |  | | 21(43.8) | | 2.01(0.94-4.33) | 0.07 |  | 5(25) | 0.82(0.25-2.69) | 0.75 |
|  | [50-85) | | 9(2.6) | | 10(2.7) | | 0.94(0.37-2.42) | | | 0.9 | |  | | 2(4.2) | | 1.83(0.35-9.5) | 0.47 |  | 2(10) | 3.14(0.57-17.43) | 0.19 |
| Burkina Faso | ≥85 | | 0(0) | | 0(0) | | n.c | | | n.c. | |  | | 0(0) | | n.c | n.c. |  | 0(0) | n.c | n.c. |
| Ghana (Noguchi) | 0 | | 18(20.2) | | 86(29.7) | | - | | | ***0.04*** | |  | | 3(27.3) | | - | ***0.17*** |  | 30(32.6) | - | ***6.43E-04*** |
| Ghana (Noguchi) | (0-25) | | 38(42.7) | | 101(34.8) | | 0.55(0.29-1.06) | | | 0.07 | |  | | 4(36.4) | | 0.56(0.11-2.88) | 0.49 |  | 27(29.3) | 0.38(0.17-0.86) | 0.02 |
|  | [25-50) | | 32(36) | | 89(30.7) | | 0.59(0.3-1.15) | | | 0.12 | |  | | 3(27.3) | | 0.47(0.08-2.72) | 0.4 |  | 26(28.3) | 0.48(0.21-1.09) | 0.08 |
|  | [50-85) | | 1(1.1) | | 14(4.8) | | 4.65(0.49-43.84) | | | 0.18 | |  | | 1(9.1) | | n.c | n.c. |  | 9(9.8) | 18.98(1.07-335.93) | 0.04 |
| Ghana (Noguchi) | ≥85 | | 0(0) | | 0(0) | | n.c | | | n.c. | |  | | 0(0) | | n.c | n.c. |  | 0(0) | n.c | n.c. |
| Ghana (Kumasi) | 0 | | 324(32.8) | | 219(31.6) | | - | | | ***0.9*** | |  | | 45(39.5) | | - | ***0.49*** |  | 69(26.6) | - | ***0.12*** |
| Ghana (Kumasi) | (0-25) | | 319(32.3) | | 228(32.9) | | 1.04(0.81-1.35) | | | 0.75 | |  | | 39(34.2) | | 0.86(0.52-1.43) | 0.56 |  | 96(37.1) | 1.53(1.03-2.26) | 0.03 |
|  | [25-50) | | 311(31.5) | | 217(31.4) | | 1.07(0.82-1.38) | | | 0.63 | |  | | 28(24.6) | | 0.68(0.4-1.17) | 0.16 |  | 81(31.3) | 1.45(0.98-2.15) | 0.07 |
|  | [50-85) | | 33(3.3) | | 28(4) | | 1.22(0.69-2.16) | | | 0.5 | |  | | 2(1.8) | | 0.54(0.12-2.37) | 0.42 |  | 13(5) | 1.76(0.78-3.97) | 0.18 |
| ¥ | ≥85 | | 0(0) | | 0(0) | | n.c | | | n.c. | |  | | 0(0) | | n.c | n.c. |  | 0(0) | n.c | n.c. |
| Nigeria | 0 | | 3(13.6) | | 13(41.9) | | - | | | ***0.04*** | |  | | 1(50) | | - | ***0.61*** |  | 2(50) | - | ***0.06*** |
| Nigeria | (0-25) | | 4(18.2) | | 8(25.8) | | 0.47(0.08-2.75) | | | 0.4 | |  | | 0(0) | | n.c | n.c. |  | 0(0) | n.c | n.c. |
|  | [25-50) | | 13(59.1) | | 7(22.6) | | 0.13(0.03-0.6) | | | 9.57E-03 | |  | | 1(50) | | 0.3(0.01-6.38) | 0.44 |  | 1(25) | n.c | n.c. |
|  | [50-85) | | 2(9.1) | | 3(9.7) | | 0.36(0.04-3.36) | | | 0.37 | |  | | 0(0) | | n.c | n.c. |  | 1(25) | 1.5(0.06-40.63) | 0.81 |
| Nigeria | ≥85 | | 0(0) | | 0(0) | | n.c | | | n.c. | |  | | 0(0) | | n.c | n.c. |  | 0(0) | n.c | n.c. |
| Cameroon | 0 | | 72(45.3) | | 114(39.9) | | - | | | ***0.24*** | |  | | 9(47.4) | | - | ***0.28*** |  | 10(24.4) | - | ***0.04*** |
| Cameroon | (0-25) | | 59(37.1) | | 120(42) | | 1.55(0.98-2.43) | | | 0.06 | |  | | 9(47.4) | | 1.42(0.52-3.89) | 0.5 |  | 21(51.2) | 3.16(1.31-7.57) | 1.01E-02 |
|  | [25-50) | | 25(15.7) | | 49(17.1) | | 1.08(0.6-1.94) | | | 0.79 | |  | | 1(5.3) | | 0.27(0.03-2.31) | 0.23 |  | 8(19.5) | 2.13(0.72-6.28) | 0.17 |
|  | [50-85) | | 3(1.9) | | 3(1) | | 0.66(0.12-3.7) | | | 0.63 | |  | | 0(0) | | n.c | n.c. |  | 2(4.9) | 5.32(0.68-41.52) | 0.11 |
| Cameroon | ≥85 | | 0(0) | | 0(0) | | n.c | | | n.c. | |  | | 0(0) | | n.c | n.c. |  | 0(0) | n.c | n.c. |
| Kenya | 0 | | 666(34.2) | | 392(36) | | - | | | ***0.2*** | |  | | 152(34.4) | | - | ***0.67*** |  | 28(38.9) | - | ***0.76*** |
| Kenya | (0-25) | | 584(29.9) | | 346(31.8) | | 1.01(0.84-1.22) | | | 0.89 | |  | | 137(31) | | 1.04(0.8-1.35) | 0.77 |  | 21(29.2) | 0.91(0.51-1.63) | 0.75 |
|  | [25-50) | | 634(32.5) | | 312(28.7) | | 0.84(0.69-1.02) | | | 0.07 | |  | | 135(30.5) | | 0.93(0.71-1.21) | 0.58 |  | 20(27.8) | 0.74(0.41-1.35) | 0.33 |
| Kenya | [50-85) | | 66(3.4) | | 39(3.6) | | 1.05(0.68-1.61) | | | 0.82 | |  | | 18(4.1) | | 1.29(0.73-2.27) | 0.38 |  | 3(4.2) | 1.17(0.34-3.97) | 0.8 |
|  | ≥85 | | 0(0) | | 0(0) | | n.c | | | n.c. | |  | | 0(0) | | n.c | n.c. |  | 0(0) | n.c | n.c. |
| Tanzania | 0 | | 87(34.8) | | 95(47.5) | | - | | | ***1.04E-02*** | |  | | 8(47.1) | | - | ***0.68*** |  | 42(58.3) | - | ***1.42E-03*** |
| Tanzania | (0-25) | | 67(26.8) | | 45(22.5) | | 0.63(0.38-1.04) | | | 0.07 | |  | | 3(17.6) | | 0.51(0.13-2.07) | 0.35 |  | 14(19.4) | 0.46(0.22-0.93) | 0.03 |
|  | [25-50) | | 86(34.4) | | 49(24.5) | | 0.47(0.29-0.76) | | | 2.00E-03 | |  | | 5(29.4) | | 0.56(0.17-1.81) | 0.33 |  | 13(18.1) | 0.27(0.13-0.55) | 2.89E-04 |
| Tanzania | [50-85) | | 10(4) | | 11(5.5) | | 1.16(0.43-3.13) | | | 0.77 | |  | | 1(5.9) | | 1.07(0.11-10.56) | 0.95 |  | 3(4.2) | 0.72(0.17-3.08) | 0.66 |
|  | ≥85 | | 0(0) | | 0(0) | | n.c | | | n.c. | |  | | 0(0) | | n.c | n.c. |  | 0(0) | n.c | n.c. |
| Malawi | 0 | | 447(36.2) | | 244(36.1) | | - | | | ***0.88*** | |  | | 167(38.3) | | - | ***0.69*** |  | 17(26.6) | - | ***0.42*** |
| Malawi | (0-25) | | 343(27.8) | | 197(29.2) | | 1.07(0.84-1.35) | | | 0.58 | |  | | 127(29.1) | | 1.01(0.77-1.32) | 0.96 |  | 20(31.2) | 1.56(0.81-3.03) | 0.19 |
|  | [25-50) | | 397(32.2) | | 211(31.3) | | 0.99(0.79-1.25) | | | 0.94 | |  | | 127(29.1) | | 0.87(0.67-1.14) | 0.32 |  | 24(37.5) | 1.62(0.86-3.07) | 0.14 |
| Malawi | [50-85) | | 47(3.8) | | 23(3.4) | | 0.9(0.53-1.52) | | | 0.68 | |  | | 15(3.4) | | 0.85(0.46-1.57) | 0.61 |  | 3(4.7) | 1.68(0.47-5.96) | 0.42 |
|  | ≥85 | | 0(0) | | 0(0) | | n.c | | | n.c. | |  | | 0(0) | | n.c | n.c. |  | 0(0) | n.c | n.c. |
| Vietnam | 0 | | 1113(96) | | 200(94.8) | | - | | | ***0.26*** | |  | | 46(97.9) | | - | ***0.55*** |  | 16(94.1) | - | ***0.94*** |
|  | (0-25) | | 0(0) | | 0(0) | | n.c | | | n.c. | |  | | 0(0) | | n.c | n.c. |  | 0(0) | n.c | n.c. |
|  | [25-50) | | 46(4) | | 10(4.7) | | 1.13(0.56-2.3) | | | 0.73 | |  | | 1(2.1) | | 0.57(0.08-4.26) | 0.58 |  | 1(5.9) | 1.08(0.13-8.72) | 0.94 |
| Vietnam | [50-85) | | 0(0) | | 0(0) | | n.c | | | n.c. | |  | | 0(0) | | n.c | n.c. |  | 0(0) | n.c | n.c. |
|  | ≥85 | | 0(0) | | 1(0.5) | | n.c | | | n.c. | |  | | 0(0) | | n.c | n.c. |  | 0(0) | n.c | n.c. |
| Papua New | 0 | | 102(90.3) | | 154(90.1) | | - | | | ***0.95*** | |  | | 17(89.5) | | - | ***0.86*** |  | 57(91.9) | - | ***0.65*** |
| Guinea | (0-25) | | 0(0) | | 0(0) | | n.c | | | n.c. | |  | | 0(0) | | n.c | n.c. |  | 0(0) | n.c | n.c. |
|  | [25-50) | | 10(8.8) | | 16(9.4) | | 1.09(0.47-2.51) | | | 0.84 | |  | | 2(10.5) | | 1.2(0.22-6.42) | 0.83 |  | 5(8.1) | 0.94(0.3-3) | 0.92 |
| Papua New Guinea | [50-85) | | 0(0) | | 0(0) | | n.c | | | n.c. | |  | | 0(0) | | n.c | n.c. |  | 0(0) | n.c | n.c. |
|  | ≥85 | | 1(0.9) | | 1(0.6) | | 0.71(0.04-11.54) | | | 0.81 | |  | | 0(0) | | n.c | n.c. |  | 0(0) | n.c | n.c. |
| All | 0 | | 3761(46.4) | | 2299(42.6) | | - | | | ***3.68E-03*** | |  | | 672(42.3) | | - | ***0.06*** |  | 400(39.8) | - | ***0.03*** |
| All | (0-25) | | 2132(26.3) | | 1626(30.1) | | 0.98(0.9-1.08) | | | 0.72 | |  | | 495(31.2) | | 1.02(0.89-1.17) | 0.81 |  | 306(30.4) | 1.08(0.9-1.29) | 0.41 |
|  | [25-50) | | 2001(24.7) | | 1310(24.3) | | 0.88(0.8-0.97) | | | 8.59E-03 | |  | | 377(23.7) | | 0.83(0.71-0.96) | 1.04E-02 |  | 252(25.1) | 1(0.83-1.21) | 0.99 |
|  | [50-85) | | 210(2.6) | | 156(2.9) | | 1.02(0.81-1.28) | | | 0.85 | |  | | 44(2.8) | | 0.93(0.66-1.31) | 0.67 |  | 45(4.5) | 1.71(1.16-2.53) | 6.86E-03 |
| All | ≥85 | | 2(0) | | 8(0.1) | | 7.3(1.43-37.24) | | | 0.02 | |  | | 0(0) | | n.c | n.c. |  | 2(0.2) | 9.72(1.01-93.12) | 0.05 |

Supplementary file 1G. G6PD Deficiency Score Female Categorical Model Female Association Test Results. Counts, odds ratios (ORs), 95% confidence intervals (95%CI) and P-values are presented for the effect of categories of G6PD deficiency (G6PDd) score on severe malaria and subtypes cerebral malaria and severe malarial anemia. Results are shown for all individuals combined at each site and across all sites. Results are adjusted for sickle-cell trait, ethnicity and sex. G6PDd, G6PD deficiency; n.c., not calculated.

**^a^** P-value for row for G6PDd score = 0 (shown in bold italics) is for overall model fit. P-value for rows with G6PDd score > 0 is for effect of that category of G6PDd score compared to individuals with G6PDd score = 0.

|  | G6PDd  Score | Controls | Severe Malaria | | |  | Cerebral Malaria | | |  | Severe Malarial Anemia | | |
| --- | --- | --- | --- | --- | --- | --- | --- | --- | --- | --- | --- | --- | --- |
|  |  | **N(%)** | **N(%)** | **OR (95%CI)** | ***P^a^*** |  | **N(%)** | **OR (95%CI)** | ***P^a^*** |  | **N(%)** | **OR (95%CI)** | ***P^a^*** |
| **Gambia** | 0 | 1117(67.7) | 928(70.7) | - | ***0.02*** |  | 308(74.4) | - | ***1.28E-02*** |  | 162(65.6) | - | ***0.86*** |
| Gambia | (0-25) | 338(20.5) | 280(21.3) | 1(0.82-1.2) | 0.96 |  | 76(18.4) | 0.82(0.61-1.09) | 0.17 |  | 53(21.5) | 1.06(0.75-1.48) | 0.75 |
|  | [25-50) | 0(0) | 0(0) | n.c | n.c. |  | 0(0) | n.c | n.c. |  | 0(0) | n.c | n.c. |
|  | [50-85) | 170(10.3) | 95(7.2) | 0.72(0.54-0.94) | 0.02 |  | 29(7) | 0.64(0.42-0.99) | 0.04 |  | 27(10.9) | 1.16(0.74-1.81) | 0.52 |
| Gambia | ≥85 | 24(1.5) | 9(0.7) | 0.46(0.21-1.01) | 0.05 |  | 1(0.2) | 0.15(0.02-1.15) | 0.07 |  | 5(2) | 1.38(0.51-3.74) | 0.52 |
| **Mali** | 0 | 98(58.7) | 142(58.2) | - | ***0.61*** |  | 34(73.9) | - | ***0.21*** |  | 55(53.4) | - | ***0.62*** |
| Mali | (0-25) | 37(22.2) | 54(22.1) | 1.03(0.62-1.7) | 0.91 |  | 8(17.4) | 0.65(0.27-1.6) | 0.35 |  | 23(22.3) | 1.16(0.61-2.21) | 0.65 |
|  | [25-50) | 0(0) | 0(0) | n.c | n.c. |  | 0(0) | n.c | n.c. |  | 0(0) | n.c | n.c. |
|  | [50-85) | 31(18.6) | 48(19.7) | 0.98(0.57-1.67) | 0.93 |  | 4(8.7) | 0.35(0.11-1.13) | 0.08 |  | 25(24.3) | 1.33(0.69-2.55) | 0.39 |
| Mali | ≥85 | 1(0.6) | 0(0) | n.c | n.c. |  | 0(0) | n.c | n.c. |  | 0(0) | n.c | n.c. |
| **Burkina Faso** | 0 | 217(58.5) | 255(53.3) | - | ***0.3*** |  | 34(57.6) | - | ***0.14*** |  | 9(50) | - | ***0.63*** |
| Burkina Faso | (0-25) | 103(27.8) | 150(31.4) | 1.28(0.94-1.76) | 0.12 |  | 21(35.6) | 1.38(0.76-2.5) | 0.3 |  | 5(27.8) | 1.24(0.4-3.8) | 0.71 |
|  | [25-50) | 0(0) | 0(0) | n.c | n.c. |  | 0(0) | n.c | n.c. |  | 0(0) | n.c | n.c. |
|  | [50-85) | 51(13.7) | 72(15.1) | 1.19(0.79-1.79) | 0.39 |  | 4(6.8) | 0.49(0.17-1.45) | 0.2 |  | 4(22.2) | 1.85(0.55-6.28) | 0.32 |
| Burkina Faso | ≥85 | 0(0) | 1(0.2) | n.c | n.c. |  | 0(0) | n.c | n.c. |  | 0(0) | n.c | n.c. |
| **Ghana (Noguchi)** | 0 | 71(65.7) | 205(54.2) | - | ***0.15*** |  | 8(80) | - | ***0.64*** |  | 81(53.3) | - | ***0.08*** |
| Ghana (Noguchi) | (0-25) | 22(20.4) | 88(23.3) | 1.41(0.82-2.44) | 0.22 |  | 1(10) | 0.45(0.05-3.87) | 0.47 |  | 35(23) | 1.5(0.78-2.88) | 0.22 |
|  | [25-50) | 0(0) | 0(0) | n.c | n.c. |  | 0(0) | n.c | n.c. |  | 0(0) | n.c | n.c. |
|  | [50-85) | 15(13.9) | 84(22.2) | 1.9(1.02-3.54) | 0.04 |  | 1(10) | 0.51(0.06-4.59) | 0.55 |  | 36(23.7) | 2.11(1.05-4.26) | 0.04 |
| Ghana (Noguchi) | ≥85 | 0(0) | 1(0.3) | n.c | n.c. |  | 0(0) | n.c | n.c. |  | 0(0) | n.c | n.c. |
| **Ghana (Kumasi)** | 0 | 582(56) | 445(55.8) | - | ***0.97*** |  | 70(60.3) | - | ***0.77*** |  | 169(58.5) | - | ***0.19*** |
| Ghana (Kumasi) | (0-25) | 268(25.8) | 192(24.1) | 1.02(0.81-1.3) | 0.85 |  | 28(24.1) | 0.9(0.55-1.48) | 0.68 |  | 49(17) | 0.77(0.53-1.11) | 0.16 |
|  | [25-50) | 0(0) | 0(0) | n.c | n.c. |  | 0(0) | n.c | n.c. |  | 0(0) | n.c | n.c. |
|  | [50-85) | 189(18.2) | 159(19.9) | 1.07(0.83-1.39) | 0.61 |  | 18(15.5) | 0.76(0.43-1.36) | 0.36 |  | 71(24.6) | 1.21(0.85-1.74) | 0.29 |
| ¥ | ≥85 | 1(0.1) | 1(0.1) | 0.96(0.06-16.14) | 0.98 |  | 0(0) | n.c | n.c. |  | 0(0) | n.c | n.c. |
| **Nigeria** | 0 | 10(55.6) | 35(76.1) | - | ***0.11*** |  | 2(50) | - | **n.c.** |  | 2(66.7) | - | **n.c.** |
| Nigeria | (0-25) | 5(27.8) | 7(15.2) | 0.27(0.06-1.21) | 0.09 |  | 2(50) | n.c | n.c. |  | 0(0) | n.c | n.c. |
|  | [25-50) | 0(0) | 0(0) | n.c | n.c. |  | 0(0) | n.c | n.c. |  | 0(0) | n.c | n.c. |
|  | [50-85) | 3(16.7) | 4(8.7) | 0.22(0.04-1.3) | 0.09 |  | 0(0) | n.c | n.c. |  | 1(33.3) | n.c | n.c. |
| Nigeria | ≥85 | 0(0) | 0(0) | n.c | n.c. |  | 0(0) | n.c | n.c. |  | 0(0) | n.c | n.c. |
| **Cameroon** | 0 | 273(65.5) | 212(63.1) | - | ***0.44*** |  | 13(65) | - | ***0.98*** |  | 21(51.2) | - | ***0.04*** |
| Cameroon | (0-25) | 96(23) | 79(23.5) | 1.04(0.73-1.49) | 0.82 |  | 5(25) | 1.11(0.38-3.22) | 0.85 |  | 11(26.8) | 1.43(0.64-3.18) | 0.38 |
|  | [25-50) | 0(0) | 0(0) | n.c | n.c. |  | 0(0) | n.c | n.c. |  | 0(0) | n.c | n.c. |
|  | [50-85) | 48(11.5) | 45(13.4) | 1.35(0.85-2.15) | 0.2 |  | 2(10) | 0.96(0.21-4.41) | 0.95 |  | 9(22) | 3.22(1.35-7.68) | 8.61E-03 |
| Cameroon | ≥85 | 0(0) | 0(0) | n.c | n.c. |  | 0(0) | n.c | n.c. |  | 0(0) | n.c | n.c. |
| **Kenya** | 0 | 1191(60) | 661(57.2) | - | ***0.24*** |  | 276(60.1) | - | ***0.44*** |  | 40(46.5) | - | ***0.02*** |
| Kenya | (0-25) | 416(21) | 263(22.8) | 1.13(0.93-1.36) | 0.21 |  | 109(23.7) | 1.13(0.88-1.46) | 0.34 |  | 18(20.9) | 1.25(0.71-2.21) | 0.45 |
|  | [25-50) | 0(0) | 0(0) | n.c | n.c. |  | 0(0) | n.c | n.c. |  | 0(0) | n.c | n.c. |
| Kenya | [50-85) | 377(19) | 231(20) | 1.16(0.95-1.41) | 0.14 |  | 74(16.1) | 0.89(0.67-1.18) | 0.42 |  | 28(32.6) | 2.35(1.42-3.88) | 8.79E-04 |
|  | ≥85 | 1(0.1) | 0(0) | 0(0-4.88e+162) | 0.95 |  | 0(0) | 0(0-2.42e+271) | 0.97 |  | 0(0) | n.c | n.c. |
| **Tanzania** | 0 | 130(63.4) | 143(63) | - | ***0.34*** |  | 11(64.7) | - | ***1*** |  | 69(64.5) | - | ***0.81*** |
| Tanzania | (0-25) | 35(17.1) | 51(22.5) | 1.19(0.71-1.98) | 0.51 |  | 3(17.6) | 0.97(0.25-3.83) | 0.97 |  | 22(20.6) | 1.12(0.58-2.15) | 0.74 |
|  | [25-50) | 0(0) | 0(0) | n.c | n.c. |  | 0(0) | n.c | n.c. |  | 0(0) | n.c | n.c. |
| Tanzania | [50-85) | 40(19.5) | 33(14.5) | 0.73(0.42-1.26) | 0.26 |  | 3(17.6) | 0.96(0.24-3.85) | 0.96 |  | 16(15) | 0.85(0.43-1.69) | 0.64 |
|  | ≥85 | 0(0) | 0(0) | n.c | n.c. |  | 0(0) | n.c | n.c. |  | 0(0) | n.c | n.c. |
| **Malawi** | 0 | 826(61.3) | 415(58.9) | - | ***0.44*** |  | 271(61.7) | - | ***0.41*** |  | 35(53.8) | - | ***0.24*** |
| Malawi | (0-25) | 252(18.7) | 139(19.7) | 1.15(0.9-1.47) | 0.25 |  | 89(20.3) | 1.13(0.85-1.49) | 0.4 |  | 11(16.9) | 1.08(0.54-2.16) | 0.83 |
|  | [25-50) | 0(0) | 0(0) | n.c | n.c. |  | 0(0) | n.c | n.c. |  | 0(0) | n.c | n.c. |
| Malawi | [50-85) | 270(20) | 151(21.4) | 1.11(0.88-1.4) | 0.39 |  | 79(18) | 0.89(0.67-1.18) | 0.42 |  | 19(29.2) | 1.65(0.93-2.94) | 0.09 |
|  | ≥85 | 0(0) | 0(0) | n.c | n.c. |  | 0(0) | n.c | n.c. |  | 0(0) | n.c | n.c. |
| **Vietnam** | 0 | 1326(98.5) | 574(98.6) | - | ***0.97*** |  | 161(99.4) | - | ***0.56*** |  | 12(85.7) | - | ***0.05*** |
|  | (0-25) | 0(0) | 0(0) | n.c | n.c. |  | 0(0) | n.c | n.c. |  | 0(0) | n.c | n.c. |
|  | [25-50) | 0(0) | 0(0) | n.c | n.c. |  | 0(0) | n.c | n.c. |  | 0(0) | n.c | n.c. |
| Vietnam | [50-85) | 2(0.1) | 1(0.2) | 1.14(0.1-12.6) | 0.91 |  | 0(0) | n.c | n.c. |  | 0(0) | n.c | n.c. |
|  | ≥85 | 18(1.3) | 7(1.2) | 0.9(0.37-2.18) | 0.82 |  | 1(0.6) | 0.46(0.06-3.54) | 0.46 |  | 2(14.3) | 12.28(2.48-60.82) | 2.12E-03 |
| **Papua New** | 0 | 119(92.2) | 201(94.8) | - | ***0.4*** |  | 24(96) | - | ***0.38*** |  | 52(96.3) | - | *0.42* |
| **Guinea** | (0-25) | 0(0) | 0(0) | n.c | n.c. |  | 0(0) | n.c | n.c. |  | 0(0) | n.c | n.c. |
|  | [25-50) | 0(0) | 0(0) | n.c | n.c. |  | 0(0) | n.c | n.c. |  | 0(0) | n.c | n.c. |
| Papua New Guinea | [50-85) | 0(0) | 0(0) | n.c | n.c. |  | 0(0) | n.c | n.c. |  | 0(0) | n.c | n.c. |
|  | ≥85 | 10(7.8) | 11(5.2) | 0.68(0.28-1.67) | 0.4 |  | 1(4) | 0.39(0.04-3.98) | 0.43 |  | 2(3.7) | 0.54(0.11-2.6) | 0.44 |
| **All** | 0 | 5960(67.9) | 4216(65.1) | - | ***0.08*** |  | 1212(68.4) | - | ***1.49E-03*** |  | 707(60) | - | ***1.97E-03*** |
| All | (0-25) | 1572(17.9) | 1303(20.1) | 1.08(0.99-1.19) | 0.08 |  | 342(19.3) | 1.01(0.88-1.16) | 0.86 |  | 227(19.3) | 1.05(0.87-1.25) | 0.63 |
|  | [25-50) | 0(0) | 0(0) | n.c | n.c. |  | 0(0) | n.c | n.c. |  | 0(0) | n.c | n.c. |
|  | [50-85) | 1196(13.6) | 923(14.3) | 1.05(0.95-1.16) | 0.35 |  | 214(12.1) | 0.79(0.67-0.94) | 6.31E-03 |  | 236(20) | 1.44(1.2-1.74) | 1.17E-04 |
| All | ≥85 | 55(0.6) | 30(0.5) | 0.65(0.4-1.04) | 0.07 |  | 3(0.2) | 0.26(0.08-0.85) | 0.03 |  | 9(0.8) | 1.24(0.58-2.67) | 0.58 |

Supplementary file 1H. G6PD Deficiency Score Categorical Model Male Association Test Results. Counts, odds ratios (ORs), 95% confidence intervals (95%CI) and P-values are presented for the effect of categories of G6PD deficiency (G6PDd) score on severe malaria and subtypes cerebral malaria and severe malarial anemia. Results are shown for all individuals combined at each site and across all sites. Results are adjusted for sickle-cell trait, ethnicity and sex. G6PDd, G6PD deficiency; n.c., not calculated.

**^a^** P-value for row for G6PDd score = 0 (shown in bold italics) is for overall model fit. P-value for rows with G6PDd score > 0 is for effect of that category of G6PDd score compared to individuals with G6PDd score = 0.

|  | G6PDd  Score | Controls | Severe Malaria | | |  | Cerebral Malaria | | |  | Severe Malarial Anemia | | |
| --- | --- | --- | --- | --- | --- | --- | --- | --- | --- | --- | --- | --- | --- |
|  |  | **N(%)** | **N(%)** | **OR (95%CI)** | ***P^a^*** |  | **N(%)** | **OR (95%CI)** | ***P^a^*** |  | **N(%)** | **OR (95%CI)** | ***P^a^*** |
| **Gambia** | 0 | 1883(57.4) | 1517(60.5) | - | ***8.80E-03*** |  | 510(62.6) | - | ***2.29E-04*** |  | 260(55.3) | - | ***0.48*** |
| **Gambia** | (0-25) | 858(26.2) | 683(27.3) | 1.01(0.89-1.14) | 0.92 |  | 223(27.4) | 0.97(0.81-1.17) | 0.77 |  | 128(27.2) | 1.11(0.88-1.4) | 0.39 |
|  | [25-50) | 310(9.4) | 175(7) | 0.74(0.6-0.92) | 6.86E-03 |  | 48(5.9) | 0.58(0.41-0.82) | 1.82E-03 |  | 42(8.9) | 1.06(0.73-1.54) | 0.77 |
|  | [50-85) | 205(6.2) | 116(4.6) | 0.75(0.58-0.96) | 0.02 |  | 33(4) | 0.62(0.42-0.92) | 0.02 |  | 33(7) | 1.21(0.81-1.81) | 0.35 |
| **Gambia** | ≥85 | 25(0.8) | 15(0.6) | 0.8(0.41-1.58) | 0.52 |  | 1(0.1) | 0.16(0.02-1.17) | 0.07 |  | 7(1.5) | 2.07(0.86-4.97) | 0.11 |
| **Mali** | 0 | 150(45.7) | 205(47) | - | ***0.72*** |  | 44(56.4) | - | ***0.23*** |  | 79(43.4) | - | ***0.65*** |
| **Mali** | (0-25) | 96(29.3) | 122(28) | 0.97(0.68-1.39) | 0.87 |  | 24(30.8) | 0.85(0.46-1.55) | 0.59 |  | 49(26.9) | 1.03(0.65-1.66) | 0.89 |
|  | [25-50) | 46(14) | 57(13.1) | 1(0.6-1.65) | 1 |  | 5(6.4) | 0.41(0.14-1.2) | 0.1 |  | 26(14.3) | 1.23(0.64-2.36) | 0.53 |
|  | [50-85) | 35(10.7) | 52(11.9) | 0.95(0.58-1.55) | 0.84 |  | 5(6.4) | 0.46(0.15-1.39) | 0.17 |  | 28(15.4) | 1.34(0.75-2.4) | 0.33 |
| **Mali** | ≥85 | 1(0.3) | 0(0) | n.c | n.c. |  | 0(0) | n.c | n.c. |  | 0(0) | n.c | n.c. |
| **Burkina Faso** | 0 | 328(45.5) | 385(45.5) | - | ***0.45*** |  | 46(43) | - | ***0.08*** |  | 16(42.1) | - | ***0.5*** |
| **Burkina Faso** | (0-25) | 242(33.6) | 260(30.7) | 0.98(0.78-1.24) | 0.86 |  | 34(31.8) | 1.15(0.7-1.9) | 0.57 |  | 11(28.9) | 0.89(0.39-2.05) | 0.79 |
|  | [25-50) | 91(12.6) | 118(13.9) | 1.28(0.9-1.81) | 0.16 |  | 21(19.6) | 2.22(1.11-4.46) | 0.02 |  | 5(13.2) | 0.89(0.28-2.77) | 0.83 |
|  | [50-85) | 60(8.3) | 82(9.7) | 1.09(0.75-1.59) | 0.64 |  | 6(5.6) | 0.64(0.26-1.6) | 0.34 |  | 6(15.8) | 2.08(0.77-5.65) | 0.15 |
| **Burkina Faso** | ≥85 | 0(0) | 1(0.1) | n.c | n.c. |  | 0(0) | n.c | n.c. |  | 0(0) | n.c | n.c. |
| **Ghana (Noguchi)** | 0 | 89(45.2) | 291(43.6) | - | ***0.15*** |  | 11(52.4) | - | ***0.54*** |  | 111(45.5) | - | ***0.02*** |
| **Ghana (Noguchi)** | (0-25) | 60(30.5) | 189(28.3) | 0.94(0.64-1.38) | 0.75 |  | 5(23.8) | 0.43(0.12-1.55) | 0.2 |  | 62(25.4) | 0.85(0.52-1.38) | 0.51 |
|  | [25-50) | 32(16.2) | 89(13.3) | 0.8(0.47-1.37) | 0.42 |  | 3(14.3) | 0.4(0.08-1.92) | 0.25 |  | 26(10.7) | 0.71(0.36-1.41) | 0.33 |
|  | [50-85) | 16(8.1) | 98(14.7) | 1.89(1.06-3.38) | 0.03 |  | 2(9.5) | 1.1(0.24-5.11) | 0.9 |  | 45(18.4) | 2.33(1.21-4.5) | 1.16E-02 |
| **Ghana (Noguchi)** | ≥85 | 0(0) | 1(0.1) | n.c | n.c. |  | 0(0) | n.c | n.c. |  | 0(0) | n.c | n.c. |
| **Ghana (Kumasi)** | 0 | 906(44.7) | 664(44.6) | - | ***0.95*** |  | 115(50) | - | ***0.49*** |  | 238(43.4) | - | ***0.39*** |
| **Ghana (Kumasi)** | (0-25) | 587(29) | 420(28.2) | 1.03(0.87-1.23) | 0.72 |  | 67(29.1) | 0.88(0.62-1.25) | 0.48 |  | 145(26.5) | 1.05(0.82-1.36) | 0.68 |
|  | [25-50) | 311(15.3) | 217(14.6) | 1.06(0.83-1.34) | 0.65 |  | 28(12.2) | 0.69(0.42-1.16) | 0.16 |  | 81(14.8) | 1.18(0.84-1.66) | 0.34 |
|  | [50-85) | 222(11) | 187(12.6) | 1.1(0.87-1.39) | 0.44 |  | 20(8.7) | 0.72(0.42-1.23) | 0.23 |  | 84(15.3) | 1.34(0.96-1.87) | 0.08 |
| **¥** | ≥85 | 1(0) | 1(0.1) | 0.91(0.09-9.21) | 0.94 |  | 0(0) | n.c | n.c. |  | 0(0) | n.c | n.c. |
| **Nigeria** | 0 | 13(32.5) | 48(62.3) | - | ***6.59E-03*** |  | 3(50) | - | ***0.41*** |  | 4(57.1) | - | ***0.09*** |
| **Nigeria** | (0-25) | 9(22.5) | 15(19.5) | 0.38(0.12-1.22) | 0.1 |  | 2(33.3) | 0.52(0.07-3.95) | 0.53 |  | 0(0) | n.c | n.c. |
|  | [25-50) | 13(32.5) | 7(9.1) | 0.1(0.02-0.46) | 2.96E-03 |  | 1(16.7) | 0.47(0.02-10.55) | 0.63 |  | 1(14.3) | 0.14(0.01-1.63) | 0.12 |
|  | [50-85) | 5(12.5) | 7(9.1) | 0.28(0.07-1.13) | 0.07 |  | 0(0) | n.c | n.c. |  | 2(28.6) | 2.15(0.15-30.49) | 0.57 |
| **Nigeria** | ≥85 | 0(0) | 0(0) | n.c | n.c. |  | 0(0) | n.c | n.c. |  | 0(0) | n.c | n.c. |
| **Cameroon** | 0 | 345(59.9) | 326(52.4) | - | ***0.45*** |  | 22(56.4) | - | ***0.43*** |  | 31(37.8) | - | ***7.17E-03*** |
| **Cameroon** | (0-25) | 155(26.9) | 199(32) | 1.19(0.9-1.57) | 0.22 |  | 14(35.9) | 1.22(0.6-2.48) | 0.57 |  | 32(39) | 1.98(1.14-3.44) | 0.02 |
|  | [25-50) | 25(4.3) | 49(7.9) | 1.04(0.6-1.82) | 0.88 |  | 1(2.6) | 0.28(0.03-2.32) | 0.24 |  | 8(9.8) | 1.63(0.63-4.21) | 0.31 |
|  | [50-85) | 51(8.9) | 48(7.7) | 1.33(0.85-2.08) | 0.22 |  | 2(5.1) | 0.84(0.19-3.8) | 0.82 |  | 11(13.4) | 3.65(1.64-8.16) | 1.57E-03 |
| **Cameroon** | ≥85 | 0(0) | 0(0) | n.c | n.c. |  | 0(0) | n.c | n.c. |  | 0(0) | n.c | n.c. |
| **Kenya** | 0 | 1857(47.2) | 1053(46.9) | - | ***0.07*** |  | 428(47.5) | - | ***0.66*** |  | 68(43) | - | ***0.04*** |
| **Kenya** | (0-25) | 1000(25.4) | 609(27.1) | 1.07(0.94-1.22) | 0.32 |  | 246(27.3) | 1.08(0.9-1.29) | 0.44 |  | 39(24.7) | 1.09(0.72-1.65) | 0.69 |
|  | [25-50) | 634(16.1) | 312(13.9) | 0.86(0.72-1.03) | 0.1 |  | 135(15) | 0.93(0.73-1.19) | 0.56 |  | 20(12.7) | 0.83(0.46-1.47) | 0.51 |
| **Kenya** | [50-85) | 443(11.3) | 270(12) | 1.13(0.95-1.35) | 0.16 |  | 92(10.2) | 0.95(0.74-1.23) | 0.69 |  | 31(19.6) | 2.04(1.31-3.17) | 1.57E-03 |
|  | ≥85 | 1(0) | 0(0) | n.c | n.c. |  | 0(0) | n.c | n.c. |  | 0(0) | n.c | n.c. |
| **Tanzania** | 0 | 217(47.7) | 238(55.7) | - | ***0.02*** |  | 19(55.9) | - | ***0.68*** |  | 111(62) | - | ***3.98E-03*** |
| **Tanzania** | (0-25) | 102(22.4) | 96(22.5) | 0.83(0.58-1.17) | 0.28 |  | 6(17.6) | 0.6(0.22-1.64) | 0.32 |  | 36(20.1) | 0.69(0.44-1.1) | 0.12 |
|  | [25-50) | 86(18.9) | 49(11.5) | 0.5(0.31-0.79) | 2.78E-03 |  | 5(14.7) | 0.59(0.19-1.85) | 0.37 |  | 13(7.3) | 0.31(0.15-0.61) | 7.80E-04 |
| **Tanzania** | [50-85) | 50(11) | 44(10.3) | 0.78(0.49-1.25) | 0.3 |  | 4(11.8) | 0.88(0.28-2.79) | 0.83 |  | 19(10.6) | 0.75(0.41-1.37) | 0.35 |
|  | ≥85 | 0(0) | 0(0) | n.c | n.c. |  | 0(0) | n.c | n.c. |  | 0(0) | n.c | n.c. |
| **Malawi** | 0 | 1273(49.3) | 659(47.8) | - | ***0.63*** |  | 438(50.1) | - | ***0.41*** |  | 52(40.3) | - | ***0.17*** |
| **Malawi** | (0-25) | 595(23) | 336(24.3) | 1.11(0.94-1.32) | 0.22 |  | 216(24.7) | 1.06(0.87-1.29) | 0.54 |  | 31(24) | 1.31(0.83-2.07) | 0.25 |
|  | [25-50) | 397(15.4) | 211(15.3) | 1.02(0.82-1.26) | 0.88 |  | 127(14.5) | 0.89(0.69-1.15) | 0.38 |  | 24(18.6) | 1.49(0.85-2.62) | 0.16 |
| **Malawi** | [50-85) | 317(12.3) | 174(12.6) | 1.07(0.86-1.32) | 0.55 |  | 94(10.7) | 0.88(0.68-1.13) | 0.31 |  | 22(17.1) | 1.7(1.01-2.87) | 0.05 |
|  | ≥85 | 0(0) | 0(0) | n.c | n.c. |  | 0(0) | n.c | n.c. |  | 0(0) | n.c | n.c. |
| **Vietnam** | 0 | 2439(97.4) | 774(97.6) | - | ***0.96*** |  | 207(99) | - | ***0.69*** |  | 28(90.3) | - | ***0.16*** |
|  | (0-25) | 0(0) | 0(0) | n.c | n.c. |  | 0(0) | n.c | n.c. |  | 0(0) | n.c | n.c. |
|  | [25-50) | 46(1.8) | 10(1.3) | 1.21(0.6-2.46) | 0.59 |  | 1(0.5) | 0.57(0.08-4.23) | 0.58 |  | 1(3.2) | 1.17(0.14-10.11) | 0.89 |
| **Vietnam** | [50-85) | 2(0.1) | 1(0.1) | 1.16(0.1-12.8) | 0.9 |  | 0(0) | n.c | n.c. |  | 0(0) | n.c | n.c. |
|  | ≥85 | 18(0.7) | 8(1) | 1.02(0.43-2.42) | 0.96 |  | 1(0.5) | 0.48(0.06-3.65) | 0.48 |  | 2(6.5) | 10.09(1.8-56.46) | 8.54E-03 |
| **Papua New** | 0 | 221(91.3) | 355(92.7) | - | ***0.66*** |  | 41(93.2) | - | ***0.68*** |  | 109(94) | - | ***0.6*** |
| **Guinea** | (0-25) | 0(0) | 0(0) | n.c | n.c. |  | 0(0) | n.c | n.c. |  | 0(0) | n.c | n.c. |
|  | [25-50) | 10(4.1) | 16(4.2) | 1.1(0.48-2.53) | 0.83 |  | 2(4.5) | 1.19(0.26-5.38) | 0.82 |  | 5(4.3) | 0.94(0.31-2.87) | 0.92 |
| **Papua New Guinea** | [50-85) | 0(0) | 0(0) | n.c | n.c. |  | 0(0) | n.c | n.c. |  | 0(0) | n.c | n.c. |
|  | ≥85 | 11(4.5) | 12(3.1) | 0.68(0.29-1.58) | 0.37 |  | 1(2.3) | 0.41(0.08-2.13) | 0.29 |  | 2(1.7) | 0.48(0.1-2.23) | 0.35 |
| **All** | 0 | 9721(57.6) | 6515(54.9) | - | ***0.14*** |  | 1884(56.1) | - | ***2.29E-04*** |  | 1107(50.7) | - | ***1.02E-04*** |
| **All** | (0-25) | 3704(21.9) | 2929(24.7) | 1.05(0.99-1.12) | 0.12 |  | 837(24.9) | 1.03(0.93-1.13) | 0.56 |  | 533(24.4) | 1.05(0.93-1.19) | 0.45 |
|  | [25-50) | 2001(11.8) | 1310(11) | 0.94(0.86-1.03) | 0.16 |  | 377(11.2) | 0.86(0.75-0.99) | 0.04 |  | 252(11.5) | 0.95(0.8-1.14) | 0.6 |
|  | [50-85) | 1406(8.3) | 1079(9.1) | 1.02(0.93-1.12) | 0.71 |  | 258(7.7) | 0.8(0.69-0.93) | 2.95E-03 |  | 281(12.9) | 1.49(1.26-1.76) | 2.22E-06 |
| All | ≥85 | 57(0.3) | 38(0.3) | 0.87(0.55-1.36) | 0.54 |  | 3(0.1) | 0.28(0.09-0.86) | 0.03 |  | 11(0.5) | 1.44(0.66-3.17) | 0.36 |

Supplementary file 1I. G6PD Deficiency Score Categorical Model All Individuals Association Test Results. Counts, odds ratios (ORs), 95% confidence intervals (95%CI) and P-values are presented for the effect of categories of G6PD deficiency (G6PDd) score on severe malaria and subtypes cerebral malaria and severe malarial anemia. Results are shown for all individuals combined at each site and across all sites. Results are adjusted for sickle-cell trait, ethnicity and sex. G6PDd, G6PD deficiency; n.c., not calculated.

**^a^** P-value for row for G6PDd score = 0 (shown in bold italics) is for overall model fit. P-value for rows with G6PDd score > 0 is for effect of that category of G6PDd score compared to individuals with G6PDd score = 0.

| Site | Phenotype | G6PDd Score  Females | | | |  | G6PDd Score  Males | | | | |  | G6PDd Score  Females and males | | | |
| --- | --- | --- | --- | --- | --- | --- | --- | --- | --- | --- | --- | --- | --- | --- | --- | --- |
|  |  | **Count** | **Mean** | **OR (95%CI)** | ***P*** |  | **Count** | **Mean^a^** | **OR (95%CI)** | | ***P*** |  | **Count** | **Mean** | **OR (95%CI)** | ***P*** |
| Gambia | CM | 401 | 9.1 | 0.88(0.81-0.95) | 1.36E-03 |  | 414 | 8.5 | 0.92(0.86-0.97) | 4.20E-03 | |  | 815 | 8.8 | 0.9(0.86-0.95) | 2.19E-05 |
| Gambia | SMA | 223 | 13.4 | 1.06(0.97-1.15) | 0.20 |  | 247 | 13.3 | 1.03(0.97-1.09) | 0.39 | |  | 470 | 13.3 | 1.04(0.99-1.09) | 0.15 |
|  | SM | 1194 | 10.8 | 0.96(0.91-1.01) | 0.10 |  | 1312 | 9.6 | 0.95(0.91-0.98) | 5.39E-03 | |  | 2506 | 10.2 | 0.95(0.93-0.98) | 1.45E-03 |
| Gambia | CTL | 1632 | 12.2 | 1 | - |  | 1649 | 12.2 | 1 | - | |  | 3281 | 12.2 | 1 | - |
| Mali | CM | 32 | 14.1 | 0.9(0.7-1.15) | 0.40 |  | 46 | 9.1 | 0.84(0.71-1) | 0.05 | |  | 78 | 11.2 | 0.86(0.74-1) | 0.06 |
| Mali | SMA | 79 | 19.7 | 1.09(0.93-1.27) | 0.27 |  | 103 | 20.2 | 1.04(0.94-1.14) | 0.49 | |  | 182 | 20.0 | 1.05(0.97-1.14) | 0.23 |
|  | SM | 192 | 17.0 | 0.99(0.87-1.12) | 0.87 |  | 244 | 17.2 | 0.99(0.91-1.07) | 0.78 | |  | 436 | 17.1 | 0.99(0.93-1.06) | 0.83 |
| Mali | CTL | 161 | 16.6 | 1 | - |  | 167 | 17.1 | 1 | - | |  | 328 | 16.8 | 1 | - |
| Burkina Faso | CM | 48 | 22.3 | 1.2(1.02-1.41) | 0.03 |  | 59 | 11.5 | 0.93(0.81-1.07) | 0.32 | |  | 107 | 16.4 | 1.03(0.93-1.13) | 0.59 |
| Burkina Faso | SMA | 20 | 19.1 | 1.09(0.85-1.39) | 0.50 |  | 18 | 20.0 | 1.1(0.91-1.33) | 0.32 | |  | 38 | 19.5 | 1.1(0.94-1.28) | 0.25 |
|  | SM | 368 | 17.2 | 1.03(0.95-1.13) | 0.44 |  | 478 | 16.3 | 1.04(0.97-1.1) | 0.26 | |  | 846 | 16.7 | 1.04(0.98-1.09) | 0.18 |
| Burkina Faso | CTL | 350 | 16.0 | 1 | - |  | 371 | 14.5 | 1 | - | |  | 721 | 15.2 | 1 | - |
| Ghana (Noguchi) | CM | 11 | 21.1 | 1.09(0.73-1.63) | 0.69 |  | 10 | 8.5 | 0.88(0.62-1.25) | 0.48 | |  | 21 | 15.1 | 0.96(0.75-1.24) | 0.78 |
| Ghana (Noguchi) | SMA | 92 | 20.2 | 1.06(0.9-1.26) | 0.47 |  | 152 | 20.0 | 1.12(1.01-1.25) | 0.03 | |  | 244 | 20.1 | 1.1(1.01-1.2) | 0.03 |
|  | SM | 290 | 18.9 | 1.01(0.88-1.16) | 0.86 |  | 378 | 19.4 | 1.11(1.01-1.22) | 0.03 | |  | 668 | 19.2 | 1.07(1-1.16) | 0.06 |
| Ghana (Noguchi) | CTL | 89 | 19.3 | 1 | - |  | 108 | 13.1 | 1 | - | |  | 197 | 15.9 | 1 | - |
| Ghana (Kumasi) | CM | 114 | 13.7 | 0.89(0.78-1.01) | 0.07 |  | 116 | 14.9 | 0.96(0.88-1.05) | 0.34 | |  | 230 | 14.3 | 0.93(0.87-1.01) | 0.07 |
| Ghana (Kumasi) | SMA | 259 | 19.1 | 1.07(0.98-1.16) | 0.11 |  | 289 | 19.4 | 1.03(0.97-1.09) | 0.35 | |  | 548 | 19.2 | 1.04(0.99-1.09) | 0.10 |
|  | SM | 692 | 18.0 | 1.02(0.96-1.08) | 0.56 |  | 797 | 17.9 | 1.01(0.97-1.05) | 0.61 | |  | 1489 | 17.9 | 1.01(0.98-1.05) | 0.44 |
| Ghana (Kumasi) | CTL | 987 | 17.7 | 1 | - |  | 1040 | 17.1 | 1 | - | |  | 2027 | 17.4 | 1 | - |
| Nigeria | CM | 2 | 21.3 | 0.78(0.37-1.63) | 0.51 |  | 4 | 10.0 | n.c. | n.c. | |  | 6 | 13.8 | 0.72(0.46-1.13) | 0.16 |
| Nigeria | SMA | 4 | 24.4 | 0.9(0.5-1.61) | 0.71 |  | 3 | 21.7 | n.c. | n.c. | |  | 7 | 23.2 | 1.05(0.62-1.79) | 0.85 |
|  | SM | 31 | 18.1 | 0.74(0.56-0.98) | 0.04 |  | 46 | 8.7 | 0.78(0.6-1.02) | 0.07 | |  | 77 | 12.5 | 0.77(0.63-0.94) | 8.47E-03 |
| Nigeria | CTL | 22 | 31.0 | 1 | - |  | 18 | 16.4 | 1 | - | |  | 40 | 24.4 | 1 | - |
| Cameroon | CM | 19 | 8.0 | 0.81(0.55-1.2) | 0.30 |  | 20 | 11.5 | 1(0.8-1.25) | 0.99 | |  | 39 | 9.8 | 0.94(0.77-1.15) | 0.56 |
| Cameroon | SMA | 41 | 17.6 | 1.24(1-1.52) | 0.04 |  | 41 | 19.6 | 1.2(1.05-1.37) | 8.58E-03 | |  | 82 | 18.6 | 1.21(1.08-1.35) | 9.35E-04 |
|  | SM | 286 | 12.3 | 1.02(0.89-1.18) | 0.74 |  | 336 | 13.4 | 1.05(0.97-1.12) | 0.21 | |  | 622 | 12.9 | 1.04(0.98-1.11) | 0.20 |
| Cameroon | CTL | 159 | 11.3 | 1 | - |  | 417 | 12.1 | 1 | - | |  | 576 | 11.9 | 1 | - |
| Kenya | CM | 442 | 17.7 | 1(0.94-1.06) | 0.97 |  | 459 | 15.2 | 0.98(0.94-1.03) | 0.46 | |  | 901 | 16.4 | 0.99(0.96-1.03) | 0.58 |
| Kenya | SMA | 72 | 15.9 | 0.94(0.82-1.08) | 0.38 |  | 86 | 25.3 | 1.14(1.06-1.23) | 8.78E-04 | |  | 158 | 21.0 | 1.08(1.01-1.16) | 0.03 |
|  | SM | 1089 | 16.8 | 0.98(0.94-1.02) | 0.27 |  | 1155 | 17.6 | 1.02(0.99-1.05) | 0.15 | |  | 2244 | 17.2 | 1.01(0.98-1.03) | 0.59 |
| Kenya | CTL | 1950 | 17.7 | 1 | - |  | 1985 | 16.6 | 1 | - | |  | 3935 | 17.2 | 1 | - |
| Tanzania | CM | 17 | 15.1 | 0.89(0.67-1.19) | 0.42 |  | 17 | 15.0 | 0.99(0.8-1.23) | 0.95 | |  | 34 | 15.1 | 0.94(0.79-1.13) | 0.54 |
| Tanzania | SMA | 72 | 11.4 | 0.78(0.66-0.93) | 0.00 |  | 107 | 13.8 | 0.98(0.88-1.09) | 0.67 | |  | 179 | 12.8 | 0.91(0.83-1) | 0.04 |
|  | SM | 200 | 15.2 | 0.92(0.82-1.02) | 0.10 |  | 227 | 13.9 | 0.96(0.88-1.04) | 0.31 | |  | 427 | 14.5 | 0.94(0.88-1) | 0.06 |
| Tanzania | CTL | 250 | 17.9 | 1 | - |  | 205 | 16.1 | 1 | - | |  | 455 | 17.1 | 1 | - |
| Malawi | CM | 436 | 16.3 | 0.97(0.91-1.03) | 0.30 |  | 439 | 15.8 | 0.98(0.94-1.03) | 0.47 | |  | 875 | 16.0 | 0.98(0.94-1.01) | 0.23 |
| Malawi | SMA | 64 | 20.7 | 1.1(0.97-1.25) | 0.15 |  | 65 | 22.4 | 1.08(0.99-1.18) | 0.09 | |  | 129 | 21.6 | 1.09(1.01-1.17) | 0.03 |
|  | SM | 675 | 17.0 | 0.99(0.94-1.04) | 0.67 |  | 705 | 17.9 | 1.02(0.98-1.05) | 0.36 | |  | 1380 | 17.4 | 1.01(0.98-1.04) | 0.61 |
| Malawi | CTL | 1234 | 17.5 | 1 | - |  | 1348 | 16.8 | 1 | - | |  | 2582 | 17.1 | 1 | - |
| Vietnam | CM | 47 | 1.0 | 0.89(0.58-1.37) | 0.60 |  | 162 | 0.6 | 0.91(0.74-1.13) | 0.41 | |  | 209 | 0.7 | 0.91(0.75-1.11) | 0.36 |
| Vietnam | SMA | 17 | 2.8 | 1.02(0.65-1.59) | 0.93 |  | 14 | 13.6 | 1.3(1.1-1.54) | 2.50E-03 | |  | 31 | 7.7 | 1.23(1.02-1.48) | 0.03 |
|  | SM | 211 | 2.7 | 1.06(0.93-1.22) | 0.39 |  | 582 | 1.3 | 0.99(0.91-1.08) | 0.84 | |  | 793 | 1.6 | 1.01(0.94-1.09) | 0.73 |
| Vietnam | CTL | 1159 | 1.8 | 1 | - |  | 1346 | 1.4 | 1 | - | |  | 2505 | 1.6 | 1 | - |
| Papua New Guinea | CM | 19 | 5.0 | 1(0.72-1.38) | 0.99 |  | 25 | 3.8 | 0.91(0.71-1.16) | 0.43 | |  | 44 | 4.3 | 0.94(0.82-1.08) | 0.40 |
| Papua New Guinea | SMA | 62 | 3.8 | 0.95(0.76-1.19) | 0.67 |  | 54 | 3.5 | 0.94(0.79-1.11) | 0.44 | |  | 116 | 3.7 | 0.94(0.83-1.07) | 0.36 |
|  | SM | 171 | 5.0 | 1(0.86-1.17) | 0.96 |  | 212 | 4.9 | 0.96(0.87-1.06) | 0.40 | |  | 383 | 5.0 | 0.97(0.9-1.05) | 0.49 |
| Papua New Guinea | CTL | 113 | 5.0 | 1 | - |  | 129 | 7.4 | 1 | - | |  | 242 | 6.3 | 1 | - |
| All | CM | 1588 | 14.1 | 0.96(0.93-0.99) | 1.43E-02 |  | 1771 | 11.9 | 0.96(0.94-0.99) | 1.60E-03 | |  | 3359 | 13.0 | 0.96(0.94-0.98) | 5.61E-05 |
| All | SMA | 1005 | 16.0 | 1.03(0.99-1.08) | 0.12 |  | 1179 | 17.6 | 1.05(1.03-1.08) | 1.78E-04 | |  | 2184 | 16.9 | 1.05(1.02-1.07) | 1.35E-04 |
|  | SM | 5399 | 14.6 | 0.98(0.96-1.01) | 0.15 |  | 6472 | 13.7 | 1(0.99-1.02) | 0.61 | |  | 11871 | 14.1 | 1(0.98-1.01) | 0.64 |
| All | CTL | 8106 | 14.0 | 1 | - |  | 8783 | 13.0 | 1 | - | |  | 16889 | 13.5 | 1 | - |

Supplementary file 1J. G6PD Deficiency Score Additive Model Association Test Results. Counts, mean G6PDd score and odds ratios (ORs), 95% confidence intervals (95%CI) and *P*-values are presented for the effect of each 10% increase in the G6PD deficiency score on severe malaria and subtypes cerebral malaria and severe malarial anemia. Results are shown for females, males and all individuals combined at each site and across all sites. Results assume a linear relationship between the log-odds of disease and G6PDd score and are adjusted for sickle-cell trait, ethnicity and sex. CM, cerebral malaria; CTL, control; SM; severe malaria; SMA, severe malarial anemia; n.c., not calculated.
